# Association of COVID-19 with acute and post-acute risk of multiple different complications and mortality in patients infected with omicron variant stratified by initial disease severity: a cohort study in Hong Kong

**DOI:** 10.1186/s12916-024-03630-6

**Published:** 2024-10-14

**Authors:** Eric Yuk Fai Wan, Ran Zhang, Sukriti Mathur, Vincent Ka Chun Yan, Francisco Tsz Tsun Lai, Celine Sze Ling Chui, Xue Li, Carlos King Ho Wong, Esther Wai Yin Chan, Chak Sing Lau, Ian Chi Kei Wong

**Affiliations:** 1 Centre for Safe Medication Practice and Research, Department of Pharmacology and Pharmacy, Li Ka Shing Faculty of Medicine, The University of Hong Kong, L02-57 2/F, Laboratory Block, 21 Sassoon Road, Pokfulam, Hong Kong, SAR China; 2 Laboratory of Data Discovery for Health (D24H), Hong Kong Science and Technology Park, Hong Kong, China; 3 Department of Family Medicine and Primary Care, School of Clinical Medicine, Li Ka Shing Faculty of Medicine, The University of Hong Kong, Hong Kong, China; 4 School of Nursing, Li Ka Shing Faculty of Medicine, The University of Hong Kong, Hong Kong, China; 5 School of Public Health, Li Ka Shing Faculty of Medicine, The University of Hong Kong, Hong Kong, China; 6 Department of Medicine, School of Clinical Medicine, Li Ka Shing Faculty of Medicine, The University of Hong Kong`, Hong Kong, China; 7 Research Department of Practice and Policy, School of Pharmacy, University College London, London, UK; 8 Department of Pharmacy, The University of Hong Kong-Shenzhen Hospital, Shenzhen, China; 9 The University of Hong Kong Shenzhen Institute of Research and Innovation, Shenzhen, China; 10 Aston Pharmacy School, Aston University, Birmingham, B4 7ET UK

**Keywords:** COVID-19, Severity, Multiple organ complications, Age-specific association, PASC

## Abstract

**Background:**

Few studies have attempted to use clinical and laboratory parameters to stratify COVID-19 patients with severe versus non-severe initial disease and evaluate age-specific differences in developing multiple different COVID-19-associated disease outcomes.

**Methods:**

A retrospective cohort included patients from the electronic health database of Hong Kong Hospital Authority between 1 January 2022 and 15 August 2022 until 15 November 2022. The cohort was divided into three cohorts by age (≤ 40, 41–64, and ≥ 65 years old). Each age cohort was stratified into four groups: (1) COVID-19 critically exposed group (ICU admission, mechanical ventilation support, CRP > 80 mg/L, or D-dimer > 2 g/mL), (2) severely exposed group (CRP 30–80 mg/L, D-dimer 0.5–2 g/mL, or CT value < 20), (3) mildly–moderately exposed group (COVID-19 positive-tested but not fulfilling the criteria for the aforementioned critically and severely exposed groups), and (4) unexposed group (without COVID-19). The characteristics between groups were adjusted with propensity score-based marginal mean weighting through stratification. Cox regression was conducted to determine the association of COVID-19 disease severity with disease outcomes and mortality in the acute and post-acute phase (< 30 and ≥ 30 days from COVID-19 infection) in each age group.

**Results:**

A total of 286,114, 320,304 and 194,227 patients with mild–moderate COVID-19 infection; 18,419, 23,678 and 31,505 patients with severe COVID-19 infection; 1,168, 2,261 and 10,178 patients with critical COVID-19 infection, and 1,143,510, 1,369,365 and 1,012,177 uninfected people were identified in aged ≤ 40, 40–64, and ≥ 65 groups, respectively. Compared to the unexposed group, a general trend tending towards an increase in risks of multiple different disease outcomes as COVID-19 disease severity increases, with advancing age, was identified in both the acute and post-acute phases. Notably, the mildly–moderately exposed group were associated with either insignificant risks (aged ≤ 40) or the lowest risks (aged > 40) for the disease outcomes in the acute phase of infection (e.g., mortality risk HR (aged ≤ 40): 1.0 (95%CI: 0.5,2.0), HR (aged 41–64): 2.1 (95%CI: 1.8, 2.6), HR (aged > 65): 4.8 (95%CI: 4.6, 5.1)); while in the post-acute phase, these risks were largely insignificant in those aged < 65, remaining significant only in the elderly (age ≥ 65) (e.g., mortality risk HR (aged ≤ 40): 0.8 (95%CI: (0.5, 1.0)), HR (aged 41–64): 1.1 (95%CI: 1.0,1.2), HR (aged > 65): 1.5 (95%CI: 1.5,1.6)). Fully vaccinated patients were associated with lower risks of disease outcomes than those receiving less than two doses of vaccination.

**Conclusions:**

The risk of multiple different disease outcomes in both acute and post-acute phases increased significantly with the increasing severity of acute COVID-19 illness, specifically among the elderly. Moreover, future studies could improve by risk-stratifying patients based on universally accepted thresholds for clinical parameters, particularly biomarkers, using biological evidence from immunological studies.

**Supplementary Information:**

The online version contains supplementary material available at 10.1186/s12916-024-03630-6.

## Background

SARS-CoV-2 infection leading to COVID-19 and the subsequent persistence of symptoms in an estimated 10% of infected patients beyond initial illness is recognized as “Post-acute sequelae SARS-CoV-2” infection (PASC) or “long COVID” [1, 2]. Previously, we have described the association of COVID-19 with increased risk of multiple different disease outcomes [3]. The exact pathophysiology of COVID-19 and its consequent long-term sequelae is not yet understood but is believed to be multifactorial, and different lines of probable mechanisms have been summarized by systematic reviews and meta-analyses consolidating extensive research from several studies [4, 5].

With the preliminary observations of severe COVID-19 symptoms being more prevalent in certain patient populations [6–10], public health experts have emphasized the importance of developing diagnostic tools such as clinical parameters and biomarkers to enable stratification of the infected patient population into groups in a graded fashion from lowest to highest risk of severe disease progression [11]. Indeed, prior small-scale clinical studies in hospitalized infected patients have assessed the accuracy of using elevated levels of biomarkers such as the inflammatory coagulation biomarkers—D-dimer and C-reactive protein (CRP)—in predicting COVID-19 prognosis and mortality risk [12, 13]. Further, requirement of intensive care unit admission and/or requiring supplemental oxygen or mechanical ventilation support have been associated with poor disease prognosis/disease severity and mortality in infected patients [14], especially in vulnerable patient populations such as the elderly and the immunocompromised [15, 16]. Evidence on the dynamic increase in viral load (corresponding to lower cycle threshold (CT) values of real-time polymerase chain reaction (PCR) test) in patients after initial infection has also been associated with higher risk of disease severity and/or mortality by few studies [17, 18]. Moreover, some studies have observed post-COVID sequelae or “long COVID” to be more prevalent in patients with higher severity of initial acute-phase disease[19, 20]. Hence, using clinical parameters including biomarkers as diagnostic tools to stratify COVID-19 patient population into subgroups by progressive increase in relative risks of developing multiple different disease outcomes may enable tailoring of treatment strategies to the needs of patients in each of these subgroups per the associated patient risk for developing the different outcomes. Thereby, vulnerable groups particularly at higher relative risks of developing persistent, long-term severe disease outcomes from COVID-19 may benefit from early identification through screening by diagnostic clinical parameters to receive additional monitoring and/or specialized multispecialty follow-up care post initial infection to minimize future possibility of morbidity and mortality [21–23].

While some studies have assessed the accuracy of potential biomarkers in discriminating between severe versus non-severe COVID-19, the scope of such evidence is limited to predict outcomes of mortality, disease prognosis/hospitalization, and/or prevalence of clinical characteristics and symptoms (rather than outcomes) in hospitalized patients diagnosed with severe acute COVID-19 [13, 24], further impeded by small sample sizes and short follow-up periods [25]. Moreover, even fewer studies have attempted to evaluate age-specific differences in developing COVID-19-associated disease outcomes in patients with severe versus non-severe initial disease [11, 26], usually choosing to solely use composite end points such as intensive care admission/oxygen support diagnostic hospital codes/records to identify patients with severe COVID-19 rather than using biomarkers as clinical parameters to identify progressively susceptible immune profiles to severe disease-outcomes in patients [27, 28], including our previous efforts.

Hence, this retrospective study aims to add to the grossly limited body of evidence presently supporting the identification of patients more likely to be impacted by short- and long-term effects of COVID-19 by the addition of three biomarkers (D-dimer and CRP biomarkers, and viral load (CT value)) to the usual clinical parameter of hospitalization/oxygen support records. By subgrouping patients by age and stratifying them into groups of progressive initial disease severity, this study evaluates the independent association of each of the stratified patient groups with the short- and long-term incident risks of developing various COVID-19-associated disease outcomes, in the acute and post-acute phase (follow-up period of up to 28 months). The main objective of this study is to test the hypothesis of a positive association of initial COVID-19 severity with the development of multiple different organ complications in the short- and long-term.

## Methods

### Study design and population

Medical records of all participants were extracted from the electronic health database of Hong Kong Hospital Authority (HA), including the medicine records of all inpatient and the majority of outpatient services. The COVID-19 vaccination records provided by the Department of Health (DH) were linked with the data extracted from the HA using the participants’ unique Hong Kong Identity Card Numbers or other personal identification numbers. The events and causes of mortality were identified using the death records obtained from the Hong Kong Deaths Registry.

In order to obtain the COVID-19 severity of Omicron variants, the inclusion period of this study was defined from 1 January 2022 to 15 August 2022, corresponding to the largest outbreak infections in Hong Kong during the omicron-dominant fifth wave of infections. Participants testing positive for COVID-19 by PCR test or rapid antigen test (RAT) during the inclusion period were identified as infected COVID-19 patients (exposed group). Subsequently, these COVID-19-positive patients were stratified into patient cohorts by initial disease-severity based on clinical parameters according to the following classification based on cutoff values of thresholds determined from prior studies [29–33]: (1) COVID-19 patients with records of ICU admission, or mechanical ventilation support, or CRP (mg/L) > 80 or D-dimer: > 2 g/mL within 7 days after COVID-19 infection were recruited in the critically exposed group cohort; (2) patients with any record of D-dimer greater than 0.5 g/mL and less than 2 g/mL, or CRP (mg/L) range between 30 to 80 within 7 days after infection, or COVID-19 infection with CT value < 20 within 3 days before or after infection were identified as severely exposed group; and (3) COVID-19 patients with records of CRP (mg/L) range between 5 and 30 within 7 days after infection, or COVID-19 infection with CT value ≥ 20 within 3 days before or after infection, and those patients with no records eligible for recruitment to the aforementioned critical and severe case cohorts, were included in the mild–moderate case cohort. The type of treatments included under the definition of mechanical ventilation support identified by International Classification of Diseases, Ninth Revision, Clinical Modification (ICD-9-CM) codes is summarized in supplementary Table 2. Further, participants in each of the three patient cohorts classified by initial disease severity were sub-grouped by age into three age groups: ≤ 40, 41–64, and ≥ 65 to assess the combinatorial effect of age and disease severity of COVID-19 infection on the outcomes.

To evaluate the short- and long-term effects of COVID-19 infection, the observation period of the study was divided into two phases: acute phase and post-acute phase. The index date was defined as the date of the first positive date of testing for COVID-19 infection for the acute phase, while the index date for the post-acute phase was defined as 30 days after the first positive testing date for COVID-19 infection (based on previous studies) [28, 34, 35].

Participants without any COVID-19 positive-testing records until 15 August 2022 were treated as the unexposed group. To ensure similar baseline characteristics of age and sex between the exposed and unexposed groups and to assign the index date for individuals without COVID-19 infection, participants in the unexposed groups were randomly selected and matched to participants in the exposed groups based on the distribution of age and sex. In addition, each of the matched unexposed participants was further assigned to a corresponding exposed patient with identical index dates to ensure similar follow-up periods. All participants in the study were followed up until the first onset of any of the outcomes of interest, mortality, or 15 November 2022, whichever occurred first.

### Definition of COVID-19 infection

In this study, COVID-19 infection was identified by either a RAT or a PCR test positive.

### Outcome measurements

The outcomes of this study included (1) major cardiovascular disease (major CVD): the composite outcome of heart failure, stroke, and coronary heart disease (CHD); (2) heart failure; (3) carditis; (4) stroke; (5) atrial fibrillation (6) flutter; (7) CHD; (8) acute coronary syndrome (ACS); (9) deep vein thrombosis (DVT); (10) chronic obstructive pulmonary diseases (COPD) and allied conditions; (11) severe liver disease; (12) pancreatitis; (13) end-stage renal disease (ESRD); (14) acute kidney disease (AKD); and (15) all-cause mortality.

The ICD-9-CM diagnostic codes used to identify all outcomes and the disease definition are listed in supplementary Table 2.

### Baseline characteristics

The baseline characteristics included age, sex, Charlson’s comorbidity index (CCI) [36], the latest vaccination status, and drug prescription before the index date. The definition of Charlson Comorbidities and other baseline characteristics were listed in the supplementary Table 2.

### Statistical analysis

Fine and stratification weights were applied to adjust the selection bias among control groups and different severity groups. As an extension method based on the propensity score stratification, this method can create the fine stratum using the propensity score on the basis of the fixed quantiles so that the extreme weights can be avoided when there is any skewed distribution of propensity score or low exposure prevalence. The fine stratification weights were conducted with 50th quantile categories of propensity score based on age, sex, CCI score, and the latest dose status. For acute and post-acute phases, the incidence rates and their corresponding 95% confidence intervals (CIs) were assessed on the basis of their Poisson distribution. The standardized mean difference (SMD) among the four groups (unexposed, mildly–moderately exposed group, severely exposed group, and critically exposed group) of < 0.1 was deemed as sufficient balance. The participants with a history of each outcome were excluded from the corresponding analysis. Cox proportional hazard regression was conducted to evaluate the association between severity and outcomes compared to the unexposed group.

Subgroup analyses stratifying patients based on sex (female and male) and the latest vaccination status (< 2 doses, ≥ 2 doses) were employed. Two sensitivity analyses were performed: (1) defined the index date as 7 days after COVID-19 infection and possible ICU admission for the analysis in the acute phase and (2) the Bonferroni correction was conducted to counteract the multiple comparisons problem. All analyses were performed by R version 4.1.2 (R Foundation for Statistical Computing, Vienna, Austria). Two-tailed tests were employed for all analyses in this study, and results with a *p*-value < 0.05 were considered as statistically significant.

## Results 

**Fig. 1:**
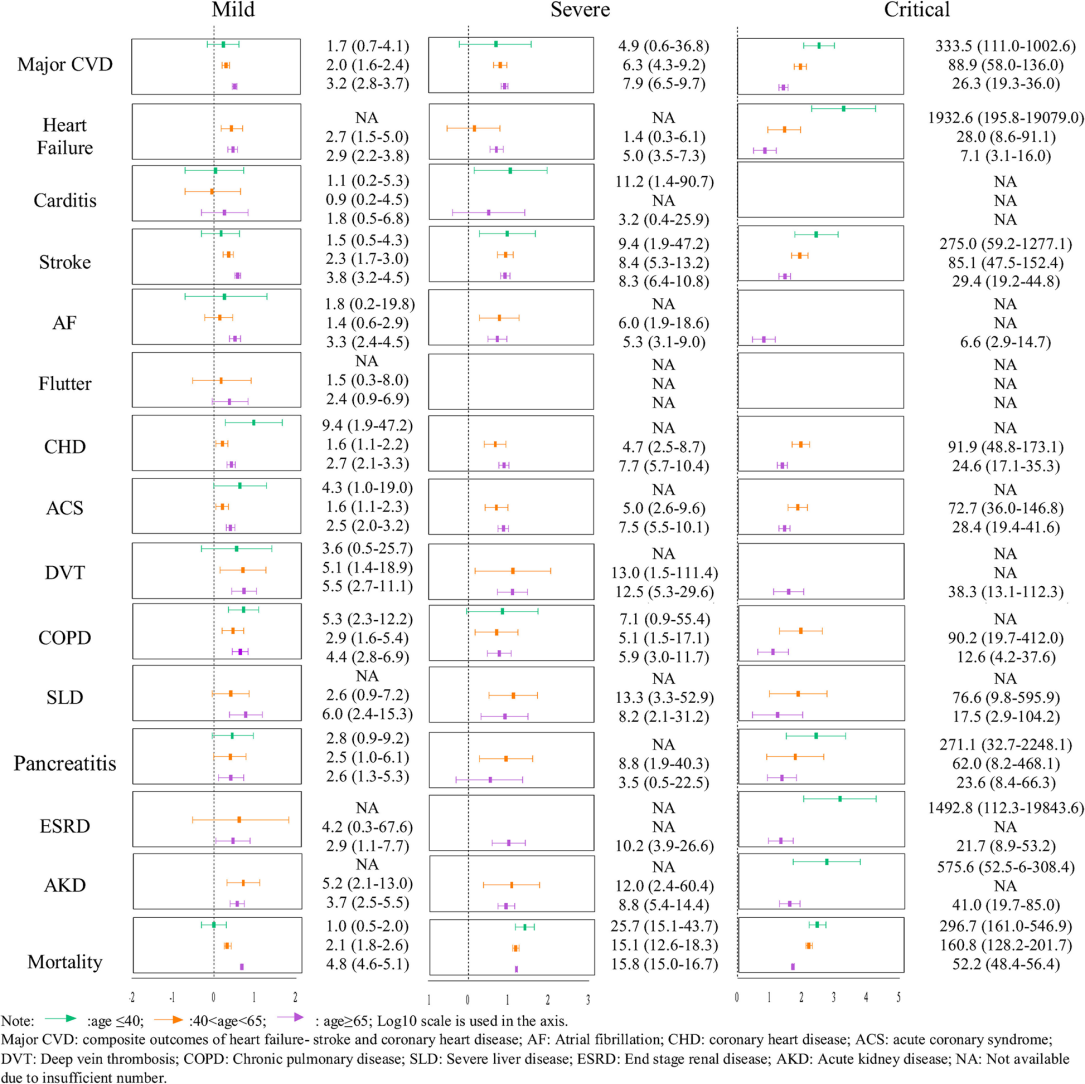
Hazard ratio of acute COVID-19 composite outcomes compared to the control groups in different COVID-19 severity populations

Figure 1 and Table 1 summarizes the selection of participants stratified into three groups by initial disease severity based on the selected clinical parameters. After subsequent sub-grouping by age into those aged (1) ≤ 40, (2) between 40 and 65, and (3) aged ≥ 65, the final cohort of patients for conducting the analysis included a total of (*n* = 286,114), (*n* = 320,304), and (*n* = 194,227) patients with mild–moderate COVID-19 infection; (*n* = 18,419), (*n* = 23,678), and (*n* = 31,505) patients with severe COVID-19 infection; (*n* = 1168), (*n* = 2261), and (*n* = 10,178) patients with critical COVID-19 infection as well as (*n* = 1,143,510), (*n* = 1,369,365), and (*n* = 1,012,177) uninfected people, respectively. Table 2 summarizes the baseline characteristics with SMD after weighting, while before weighting is listed in supplementary Table 3. The SMD for most of the characteristics among the four groups was < 0.1, indicating a good balance in all characteristics between subgroups.

**Table 1:** Hazard ratio of acute COVID-19 composite outcomes compared to the non-COVID-19 group

	**Non-COVID-19 group**		**Mild**			**Severe**			**Critical**		
	**Events**	**Incidence rate** **(per 100,000 person per day)**	**Events**	**Incidence rate** **(per 100,000 person per day)**	**HR**	**Events**	**Incidence rate** **(per 100,000 person per day)**	**HR**	**Events**	**Incidence rate ** **(per 100,000 person per day)**	**HR**
**Age ≤40**											
Major CVD	16	0.05 (0.03,0.07)	7	0.08 (0.03,0.15)	1.7 (0.7,4.1)	1	0.22 (0.04,1.01)	4.9 (0.6,36.8)	5	15.30 (6.39,33.88)	333.5 (111.0,1,002.6)
Heart failure	1	309,709.55 (70,648.28,1,625,162.08)	0	NA	NA	0	NA	NA	2	603,002,167.73 (178,680,143.24,2,086,592,822.35)	1932.6 (195.8,19079.0)
Carditis	7	0.02 (0.01,0.04)	2	0.02 (0.00,0.06)	1.1 (0.2,5.3)	1	0.23 (0.04,1.01)	11.2 (1.4,90.7)	0	NA	NA
Stroke	11	3,337,640.28 (1,810,148.88,5,745,834.82)	4	4,998,950.69 (1,894,146.80,11,949,011.81)	1.5 (0.5,4.3)	2	31,540,523.00 (4,392,358.51,101,039,779.18)	9.4 (1.9,47.2)	3	920,674,448.44 (315,303,291.43,2,536,449,638.38)	275.0 (59.2,1,277.1)
AF	2	0.01 (0.00,0.02)	1	0.01 (0.00,0.04)	1.8 (0.2,19.8)	0	NA	NA	0	NA	NA
Flutter	0	NA	1	0.01 (0.00,0.04)	NA	0	NA	NA	0	NA	NA
CHD	4	0.01 (0.00,0.03)	3	0.03 (0.01,0.08)	9.4 (1.9,47.2)	0	NA	NA	0	NA	NA
ACS	3	0.01 (0.00,0.03)	3	0.04 (0.01,0.10)	4.3 (1.0,19.0)	0	NA	NA	0	NA	NA
DVT	2	0.01 (0.00,0.02)	2	0.02 (0.00,0.06)	3.6 (0.5,25.7)	0	NA	NA	0	NA	NA
COPD	10	0.03 (0.01,0.05)	13	0.16 (0.09,0.26)	5.3 (2.3,12.2)	1	0.21 (0.04,1.03)	7.1 (0.9,55.4)	0	NA	NA
SLD	0	NA	0	NA	NA	0	NA	NA	1	382,322,500.00 (69,768,050.00,1,604,894,000.00)	NA
Pancreatitis	6	0.02 (0.01,0.04)	4	0.05 (0.02,0.12)	2.8 (0.9,9.2)	0	NA	NA	2	4.91 (0.70,16.15)	271.1 (32.7,2248.1)
ESRD	1	0.00 (0.00,0.01)	0	NA	NA	0	NA	NA	1	4.06 (0.70,16.07)	1492.8 (112.3,19843.6)
AKD	2	0.01 (0.00,0.02)	0	NA	NA	0	NA	NA	1	3.49 (0.70,16.09)	575.6 (52.5,6308.4)
All-cause mortality	46	0.13 (0.10,0.18)	12	0.14 (0.08,0.24)	1.0 (0.5,2.0)	19	3.47 (2.21,5.37)	25.7 (15.1,43.7)	14	40.03 (22.04,64.01)	296.7 (161.0,546.9)
**Age (40-65)**											
Major CVD	318	0.81 (0.72,0.90)	146	1.59 (1.35,1.87)	2.0 (1.6,2.4)	34	5.09 (3.61,7.05)	6.3 (4.3,9.2)	44	72.46 (53.68,96.59)	88.9 (58.0,136.0)
Heart failure	27	0.07 (0.05,0.10)	17	0.18 (0.11,0.28)	2.7 (1.5,5.0)	1	0.10 (0.00,0.52)	1.4 (0.3,6.1)	1	1.88 (0.37,8.59)	28.0 (8.6,91.1)
Carditis	8	0.02 (0.01,0.04)	2	0.02 (0.00,0.06)	0.9 (0.2,4.5)	0	NA	NA	0	NA	NA
Stroke	170	0.42 (0.36,0.49)	91	0.96 (0.78,1.17)	2.3 (1.7,3.0)	24	3.53 (2.34,5.17)	8.4 (5.3,13.2)	23	36.18 (23.06,52.69)	85.1 (47.5,152.4)
AF	30	0.07 (0.05,0.10)	10	0.10 (0.05,0.18)	1.4 (0.6,2.9)	3	0.44 (0.16,1.25)	6.0 (1.9,18.6)	0	NA	NA
Flutter	5	0.01 (0.00,0.02)	2	0.02 (0.00,0.06)	1.5 (0.3,8.0)	0	NA	NA	0	NA	NA
CHD	143	0.36 (0.30,0.42)	52	0.56 (0.42,0.73)	1.6 (1.1,2.2)	12	1.66 (0.90,2.85)	4.7 (2.5,8.7)	21	32.98 (20.53,48.78)	91.9 (48.8,173.1)
ACS	128	0.31 (0.26,0.37)	48	0.51 (0.38,0.67)	1.6 (1.1,2.3)	11	1.58 (0.88,2.81)	5.0 (2.6,9.6)	15	22.96 (13.07,36.58)	72.7 (36.0,146.8)
DVT	5	0.01 (0.00,0.02)	6	0.06 (0.02,0.12)	5.1 (1.4,18.9)	1	0.16 (0.03,0.79)	13.0 (1.5,111.4)	0	NA	NA
COPD	24	0.06 (0.04,0.08)	16	0.17 (0.10,0.27)	2.9 (1.6,5.4)	2	0.30 (0.09,1.04)	5.1 (1.5,17.1)	3	5.30 (1.70,13.70)	90.2 (19.7,412.0)
SLD	10	0.03 (0.01,0.04)	6	0.07 (0.03,0.14)	2.6 (0.9,7.2)	2	0.33 (0.09,1.02)	13.3 (3.3,52.9)	1	1.93 (0.37,8.56)	76.6 (9.8,595.9)
Pancreatitis	15	0.04 (0.02,0.06)	9	0.09 (0.04,0.16)	2.5 (1.0,6.1)	2	0.32 (0.09,1.02)	8.8 (1.9,40.3)	1	2.27 (0.38,8.76)	62.0 (8.2,468.1)
ESRD	1	0.00 (0.00,0.01)	1	0.01 (0.00,0.04)	4.2 (0.3,67.6)	0	NA	NA	0	NA	NA
AKD	9	0.02 (0.01,0.04)	11	0.11 (0.06,0.19)	5.2 (2.1,13.0)	2	0.26 (0.03,0.79)	12.0 (2.4,60.4)	0	NA	NA
All-cause mortality	480	1.17 (1.07,1.28)	241	2.51 (2.21,2.85)	2.1 (1.8,2.6)	125	17.69 (14.83,21.04)	15.1 (12.6,18.3)	123	188.72 (158.12,224.99)	160.8 (128.2,201.7)
**Age≥65**											
Major CVD	635	2.57 (2.38,2.77)	387	8.28 (7.48,9.13)	3.2 (2.8,3.7)	149	20.44 (17.38,23.95)	7.9 (6.5,9.7)	151	69.81 (59.25,81.56)	26.3 (19.3,36.0)
Heart failure	178	0.60 (0.52,0.70)	98	1.76 (1.44,2.14)	2.9 (2.2,3.8)	27	3.04 (2.01,4.31)	5.0 (3.5,7.3)	11	4.34 (2.35,7.45)	7.1 (3.1,16.0)
Carditis	8	0.03 (0.01,0.05)	3	0.05 (0.01,0.13)	1.8 (0.5,6.8)	1	0.09 (0.00,0.40)	3.2 (0.4,25.9)	0	NA	NA
Stroke	371	1.35 (1.22,1.49)	267	5.13 (4.54,5.78)	3.8 (3.2,4.5)	92	11.32 (9.20,13.83)	8.3 (6.4,10.8)	99	41.05 (33.40,49.57)	29.4 (19.2,44.8)
AF	108	0.37 (0.31,0.45)	68	1.23 (0.96,1.55)	3.3 (2.4,4.5)	17	1.98 (1.23,3.14)	5.3 (3.1,9.0)	6	2.48 (1.09,5.06)	6.6 (2.9,14.7)
Flutter	12	0.04 (0.02,0.07)	6	0.10 (0.04,0.21)	2.4 (0.9,6.9)	0	NA	NA	0	NA	NA
CHD	253	0.92 (0.81,1.03)	128	2.43 (2.03,2.88)	2.7 (2.1,3.3)	59	7.09 (5.50,9.14)	7.7 (5.7,10.4)	58	23.06 (17.56,29.44)	24.6 (17.1,35.3)
ACS	250	0.84 (0.74,0.95)	120	2.14 (1.79,2.55)	2.5 (2.0,3.2)	57	6.38 (4.86,8.19)	7.5 (5.5,10.1)	66	24.76 (19.28,31.29)	28.4 (19.4,41.6)
DVT	16	0.05 (0.03,0.09)	17	0.30 (0.19,0.47)	5.5 (2.7,11.1)	6	0.68 (0.31,1.44)	12.5 (5.3,29.6)	6	2.09 (0.81,4.30)	38.3 (13.1,112.3)
COPD	45	0.16 (0.12,0.21)	38	0.68 (0.49,0.93)	4.4 (2.8,6.9)	8	0.92 (0.40,1.67)	5.9 (3.0,11.7)	5	1.98 (0.86,4.55)	12.6 (4.2,37.6)
SLD	9	0.03 (0.02,0.06)	11	0.19 (0.10,0.32)	6.0 (2.4,15.3)	2	0.26 (0.07,0.79)	8.2 (2.1,31.2)	2	0.58 (0.09,2.04)	17.5 (2.9,104.2)
Pancreatitis	27	0.09 (0.06,0.13)	13	0.23 (0.13,0.39)	2.6 (1.3,5.3)	3	0.31 (0.07,0.79)	3.5 (0.5,22.5)	6	2.14 (0.81,4.31)	23.6 (8.4,66.3)
ESRD	14	0.05 (0.03,0.08)	8	0.14 (0.07,0.27)	2.9 (1.1,7.7)	4	0.49 (0.18,1.12)	10.2 (3.9,26.6)	3	1.05 (0.23,2.65)	21.7 (8.9,53.2)
AKD	67	0.22 (0.17,0.28)	47	0.82 (0.61,1.08)	3.7 (2.5,5.5)	18	1.96 (1.19,3.03)	8.8 (5.4,14.4)	25	9.33 (6.09,13.43)	41.0 (19.7,85.0)
All-cause mortality	3,329	10.98 (10.61,11.36)	3,074	53.28 (51.43,55.20)	4.8 (4.6,5.1)	1,593	174.20 (165.77,182.88)	15.8 (15.0,16.7)	1,599	583.93 (555.94,613.19)	52.2 (48.4,56.4)

Note: Major CVD: composite outcomes of heart failure, stroke and coronary heart disease; AF: Atrial fibrillation; CHD: coronary heart disease ACS: Acute coronary disorder; DVT: Deep vein thrombosis; COPD: Chronic pulmonary disease; SLD: Severe liver disease; ESRD: End stage renal disease; AKD: Acute kidney disease; NA: Not available due to insufficient number

Hazard Ratio was obtained by Cox regression adjusted with weighting

**Table 2:** Baseline characteristics of patients after weighting (acute phase)

Characteristics	Non-COVID-19 group	Mild	Severe	Critical	SMD
Age ≤ 40	*N*** = **1,143,510	*N*** = **286,114	*N*** = **18,419	*N*** = **1168	
Age, years (mean (SD))	1,143,510	286,114	18,419	1168	
Sex, male (%)	25.34 (10.53)	25.35 (10.49)	24.80 (10.86)	25.49 (10.91)	0.03
Charlson Comorbidity Index (mean (SD))	539,875 (47.2)	136,109 (47.6)	8887 (48.3)	547 (46.9)	0.02
Renin-angiotensin system agents (%)	0.04 (0.27)	0.04 (0.27)	0.05 (0.32)	0.07 (0.43)	0.05
Beta blockers (%)	7578 (0.7)	2080 (0.7)	158 (0.9)	11 (1.0)	0.02
Calcium channel blockers (%)	7874 (0.7)	2080 (0.7)	164 (0.9)	9 (0.8)	0.01
Diuretics (%)	8530 (0.7)	2310 (0.8)	152 (0.8)	12 (1.1)	0.02
Nitrates (%)	1424 (0.1)	387 (0.1)	37 (0.2)	5 (0.4)	0.03
Lipid lowering agents (%)	350 (0.0)	101 (0.0)	5 (0.0)	1 (0.1)	0.02
Insulins (%)	6310 (0.6)	1717 (0.6)	118 (0.6)	10 (0.9)	0.02
Antidiabetic drugs (%)	2329 (0.2)	632 (0.2)	38 (0.2)	3 (0.3)	0.01
Oral anticoagulants (%)	5776 (0.5)	1566 (0.5)	114 (0.6)	8 (0.7)	0.02
Antiplatelets (%)	532 (0.0)	141 (0.0)	10 (0.1)	1 (0.2)	0.02
Immunosuppressants (%)	2259 (0.2)	628 (0.2)	41 (0.2)	6 (0.5)	0.03
Dose status (%)	3087 (0.3)	820 (0.3)	58 (0.3)	5 (0.5)	0.02
0					0.04
1	229,394 (20.1)	57,433 (20.1)	3673 (19.9)	209 (17.9)	
2	132,848 (11.6)	34,555 (12.1)	2176 (11.8)	133 (11.4)	
3	505,370 (44.2)	123,830 (43.3)	8120 (44.1)	537 (46.0)	
40** < **age** < **65	*N*** = **1,369,365	*N*** = **320,304	*N*** = **23,678	*N*** = **2261	
Age, years (mean (SD))	53.57 (6.93)	53.64 (6.95)	53.55 (6.84)	53.36 (7.10)	0.02
Sex, male (%)	575,618 (42.0)	134,489 (42.0)	9724 (41.1)	944 (41.7)	0.01
Charlson Comorbidity Index (mean (SD))	1.19 (1.13)	1.20 (1.16)	1.21 (1.22)	1.27 (1.25)	0.04
Renin-angiotensin system agents (%)	157,075 (11.5)	37,411 (11.7)	2792 (11.8)	270 (12.0)	0.01
Beta blockers (%)	93,585 (6.8)	22,396 (7.0)	1663 (7.0)	136 (6.0)	0.02
Calcium channel blockers (%)	215,501 (15.7)	51,135 (16.0)	3779 (16.0)	336 (14.9)	0.02
Diuretics (%)	22,933 (1.7)	5594 (1.7)	399 (1.7)	37 (1.6)	0.00
Nitrates (%)	16,294 (1.2)	3914 (1.2)	280 (1.2)	29 (1.3)	0.01
Lipid lowering agents (%)	215,854 (15.8)	51,415 (16.1)	3875 (16.4)	380 (16.8)	0.02
Insulins (%)	17,897 (1.3)	4326 (1.4)	304 (1.3)	34 (1.5)	0.01
Antidiabetic drugs (%)	124,166 (9.1)	29,528 (9.2)	2211 (9.3)	244 (10.8)	0.03
Oral anticoagulants (%)	5469 (0.4)	1317 (0.4)	105 (0.4)	9 (0.4)	0.01
Antiplatelets (%)	63,500 (4.6)	15,258 (4.8)	1111 (4.7)	99 (4.4)	0.01
Immunosuppressants (%)	7434 (0.5)	1793 (0.6)	132 (0.6)	19 (0.8)	0.02
Dose status (%)					0.04
0	143,746 (10.5)	33,797 (10.6)	2393 (10.1)	193 (8.5)	
1	89,218 (6.5)	21,089 (6.6)	1564 (6.6)	156 (6.9)	
2	533,449 (39.0)	125,354 (39.1)	9135 (38.6)	897 (39.7)	
3	602,952 (44.0)	140,064 (43.7)	10,586 (44.7)	1015 (44.9)	
Age** ≥ **65	*N*** = **1,012,177	*N*** = **194,227	*N*** = **31,505	*N*** = **10,178	
Age, years (mean (SD))	74.83 (8.01)	74.92 (8.41)	74.64 (8.32)	74.66 (8.32)	0.02
Sex, male (%)	470,941 (46.5)	90,380 (46.5)	14,914 (47.3)	4798 (47.1)	0.01
Charlson Comorbidity Index (mean (SD))	3.64 (1.53)	3.66 (1.58)	3.68 (1.52)	3.78 (1.55)	0.05
Renin-angiotensin-system agents (%)	297,828 (29.4)	58,251 (30.0)	9564 (30.4)	3413 (33.5)	0.05
Beta blockers (%)	192,042 (19.0)	37,633 (19.4)	6222 (19.8)	2175 (21.4)	0.03
Calcium channel blockers (%)	428,164 (42.3)	83,417 (42.9)	13,496 (42.8)	4503 (44.2)	0.02
Diuretics (%)	75,840 (7.5)	15,252 (7.9)	2375 (7.5)	910 (8.9)	0.03
Nitrates (%)	56,027 (5.5)	11,198 (5.8)	1872 (5.9)	615 (6.0)	0.01
Lipid lowering agents (%)	444,266 (43.9)	86,571 (44.6)	14,385 (45.7)	4973 (48.9)	0.05
Insulins (%)	35,643 (3.5)	7137 (3.7)	1122 (3.6)	465 (4.6)	0.03
Antidiabetic drugs (%)	222,778 (22.0)	43,493 (22.4)	7332 (23.3)	2617 (25.7)	0.05
Oral anticoagulants (%)	33,968 (3.4)	6801 (3.5)	1112 (3.5)	469 (4.6)	0.03
Antiplatelets (%)	196,053 (19.4)	38,780 (20.0)	6221 (19.7)	1976 (19.4)	0.01
Immunosuppressants (%)	5230 (0.5)	1048 (0.5)	186 (0.6)	82 (0.8)	0.02
Dose status (%)					0.04
0	202,958 (20.1)	39,993 (20.6)	6039 (19.2)	1904 (18.7)	
1	134,651 (13.3)	26,209 (13.5)	4274 (13.6)	1325 (13.0)	
2	348,723 (34.5)	66,031 (34.0)	11,285 (35.8)	3566 (35.0)	
3	325,845 (32.2)	61,994 (31.9)	9908 (31.4)	3382 (33.2)	

### Acute phase

Figure 1 depicts the incidence rates and hazard ratios (HRs) of each of the disease outcomes for each patient group by initial disease severity sub-grouped by age. In general, in the acute phase of infection, patients classified as mild–moderate severity were associated with the lowest risk of developing disease outcomes and mortality, progressively increasing in those classified as the severely exposed group and critically exposed group, with the highest risks associated with the latter. Moreover, an overall additional trend of graded increase in risk associated with disease outcomes and mortality with increasing age was observed upon comparing the risks in the subgroupings by age.

Specifically, patients aged ≤ 40 classified as mildly–moderately exposed group showed largely insignificant risks for most of the outcomes and mortality, barring the cardiovascular outcome of CHD (HR: 9.4(1.9, 47.2)) and respiratory disease outcomes of COPD and allied conditions (HR: 5.3(2.3, 12.2)) than the unexposed group. Meanwhile, the severely exposed group in this age group showed a substantial increase in risk associated with the aforementioned outcomes (HR(COPD): 7.1(0.9, 55.4)) and an additional risk associated with more cardiovascular outcomes (HR (carditis): 11.2(1.4,90.7); HR (stroke): 9.4(1.9, 47.2)) and all-cause mortality (HR: 25.7(15.1, 43.7)). However, those classified as the critically exposed group were associated with the highest risk of mortality (HR: 296.7(161.0, 546.9)) and at substantially higher risks of several cardiovascular outcomes, including major CVD (HR: 333.5(111.0, 1002.6)) and carditis (HR: 28.5(3.5, 231.5)); and renal outcomes (HR (acute kidney disease): 575.6(52.5, 6308.4); HR(ESRD): 1492.8(112.3, 19,843.6)).

Similarly, in those aged between 40 and 65, although the mildly–moderately exposed group were associated with increased risks of certain cardiovascular and thrombotic outcomes (HR (major CVD): 2.0(95%CI: 1.6, 2.4); HR(DVT): 5.1(95%CI: 1.4, 18.9); HR(ACS): 1.6(95%CI: 1.1, 2.3)) than the unexposed group, these were far lower than the severely and critically exposed groups. The severely exposed group showed an increase in risk of these aforementioned outcomes (HR (major CVD): 6.3(95%CI: 4.3, 9.2); HR(DVT): 13.0(95%CI: 1.5, 111.4); HR (ACS): 5.0(95%CI: 2.6, 9.6)), and an additional risk of AF (HR: 6.0(95%CI: 1.9, 18.6)) than the unexposed group. The critically exposed group was again associated with the highest risks for these outcomes in this age group, notably cardiovascular outcomes like major CVD (HR: 88.9(95%CI: 58.0, 136.0)) and ACS (HR: 72.7(95%CI: 36.0, 146.8)).

However, in the elderly cohort comprising patients aged > 65, significantly higher risks were observed in most outcomes of interest regardless of the severity of COVID-19 infection, although the risks increased in a graded fashion by severity. Notably, increased risks were associated with cardiovascular outcomes like major CVD (HR(mild–moderate): 3.2 (95%CI: 2.8, 3.7); HR(severe): 7.9(95%CI: 6.5, 9.7); HR(critical): 26.3(95%CI: 19.3, 36.0)) and DVT (HR (mild–moderate): 5.5(95%CI: 2.7,11.1); HR (severe): 12.5(95%CI: 5.3, 29.6); HR (critical): 38.3(95%CI: 13.1, 112.3)); respiratory outcomes like COPD (HR (mild–moderate): 4.4(95%CI: 2.8, 6.9); HR (severe): 5.9(95%CI: 3.0, 11.7); HR (critical): 12.6(95%CI: 4.2, 37.6)); renal and hepatic outcomes like severe liver disease (HR(mild–moderate): 6.0 (95%CI: 2.4, 15.3); HR(severe): 8.2 (95%CI: 2.1, 31.2); HR(critical): 17.5 (2.9, 104.2)), acute kidney disease (HR(mild–moderate): 3.7 (95%CI: 2.5, 5.5); HR(severe): 8.8 (95%CI: 5.4, 14.4); HR(critical): 41.0(95%CI: 19.7,85.0)) and ESRD (HR(mild–moderate): 2.9(95%CI: 1.1, 7.7); HR(severe): 10.2(95%CI: 3.9,26.6); HR(critical): 21.7(95%CI: 8.9, 53.2)); and all-cause mortality (HR(mild–moderate): 4.8 (95%CI: 4.6, 5.1); HR (severe): 15.8 (95%CI: 15.0, 16.7); HR (critical): 52.2 (95%CI: 48.4, 56.4)). The results for the sensitivity analysis (by defining the index date as seven days after COVID-19 infection and possible ICU admission) in the acute phase were mainly consistent with the main analysis.

### Post-acute phase

**Fig. 2:**
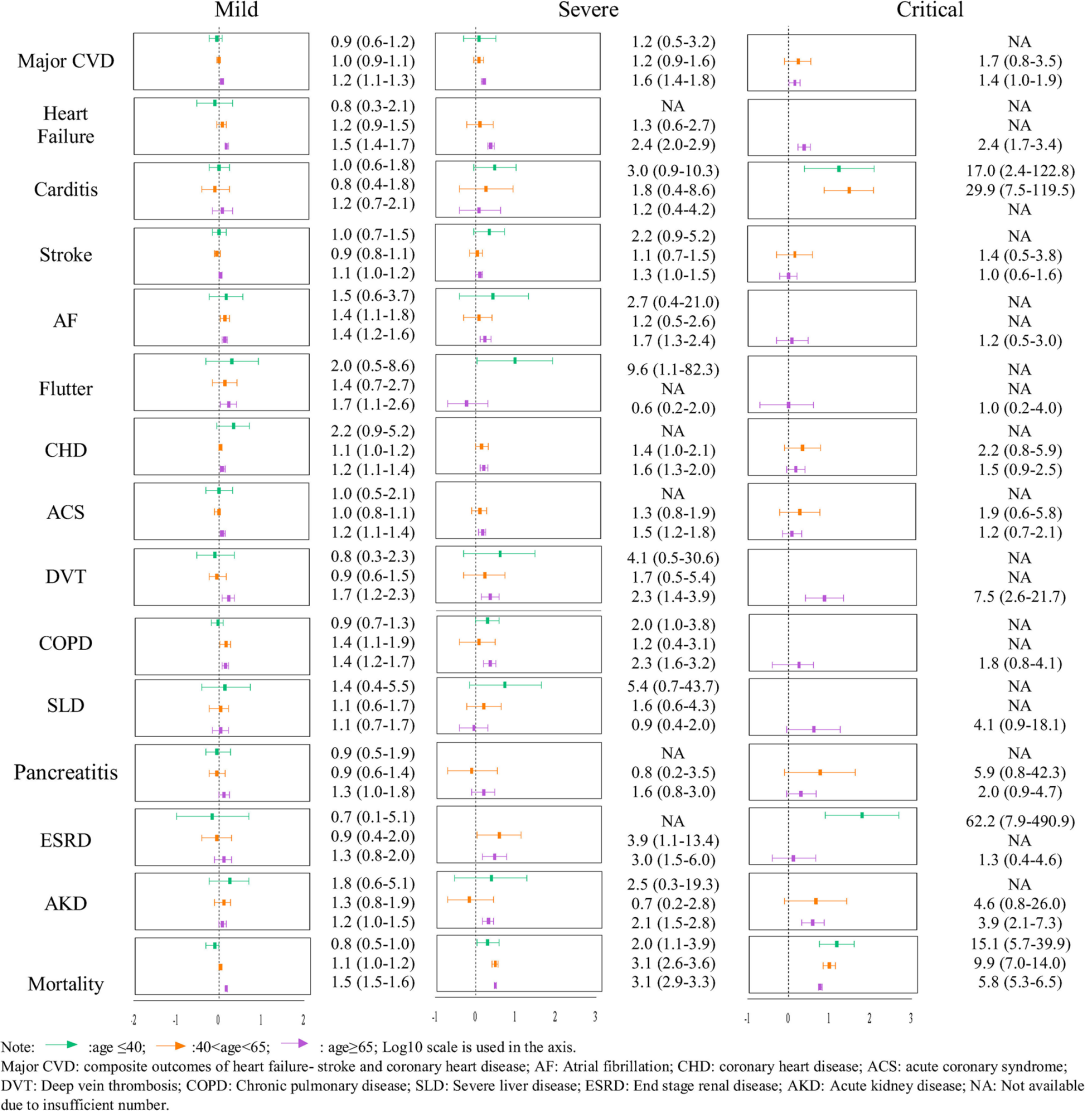
Hazard ratio of post-acute COVID-19 composite outcomes compared to the control groups in different COVID-19 severity populations

The incidence rate and HR of the outcomes in this phase among patients with and without COVID-19 infection is depicted in Table 3 and Fig. 2. The increased risk associated with most of the outcomes observed in the mildly–moderately and severely exposed groups aged under 65 years were insignificant; although participants in the severely exposed group aged under 65 were associated with significantly higher risks of all-cause mortality (HR (aged ≤ 40): 2.0(95%CI: 1.1,3.9); HR (40 < aged < 65): 3.1(95%CI: 2.6,3.6)) after COVID-19 infection in the long term.

**Table 3:** Hazard ratio of post-acute COVID-19 composite outcomes compared to the non-COVID-19 group

	**Non-COVID-19 group**		**Mild**			**Severe**			**Critical**		
	**Events**	**Incidence rate** **(per 10,000 person per year)**	**Events**	**Incidence rate** **(per 10,000 person per year)**	**HR**	**Events**	**Incidence rate** **(per 10,000 person per year)**	**HR**	**Events**	**Incidence rate ** **(per 10,000 person per year)**	**HR**
**Age≤40**											
Major CVD	180	3.1 (2.7,3.6)	39	2.7 (2.0,3.7)	0.9 (0.6,1.2)	4	3.8 (1.1,8.9)	1.2 (0.5,3.2)	0	NA	NA
Heart failure	22	0.4 (0.2,0.6)	4	0.3 (0.1,0.7)	0.8 (0.3,2.1)	0	NA	NA	0	NA	NA
Carditis	60	1.0 (0.8,1.3)	15	1.0 (0.6,1.7)	1.0 (0.6,1.8)	3	3.0 (0.6,7.3)	3.0 (0.9,10.3)	1	17.6 (4.2,95.9)	17.0 (2.4,122.8)
Stroke	115	2.0 (1.6,2.4)	30	2.1 (1.4,2.9)	1.0 (0.7,1.5)	4	4.3 (1.6,10.3)	2.2 (0.9,5.2)	0	NA	NA
AF	18	0.3 (0.2,0.5)	7	0.5 (0.2,0.9)	1.5 (0.6,3.7)	1	0.9 (0.0,3.7)	2.7 (0.4,21.0)	0	NA	NA
Flutter	5	0.1 (0.0,0.2)	3	0.2 (0.0,0.5)	2.0 (0.5,8.6)	1	0.9 (0.0,3.7)	9.6 (1.1,82.3)	0	NA	NA
CHD	50	0.9 (0.7,1.1)	9	0.6 (0.3,1.2)	2.2 (0.9,5.2)	0	NA	NA	0	NA	NA
ACS	31	0.5 (0.4,0.8)	8	0.5 (0.2,1.0)	1.0 (0.5,2.1)	0	NA	NA	0	NA	NA
DVT	18	0.3 (0.2,0.5)	4	0.2 (0.1,0.6)	0.8 (0.3,2.3)	1	1.3 (0.2,5.6)	4.1 (0.5,30.6)	0	NA	NA
COPD	231	4.0 (3.5,4.5)	54	3.7 (2.8,4.8)	0.9 (0.7,1.3)	8	7.9 (3.5,14.8)	2.0 (1.0,3.8)	0	NA	NA
SLD	8	0.1 (0.1,0.3)	3	0.2 (0.0,0.5)	1.4 (0.4,5.5)	1	0.7 (0.0,3.7)	5.4 (0.7,43.7)	0	NA	NA
Pancreatitis	42	0.7 (0.5,1.0)	10	0.7 (0.3,1.2)	0.9 (0.5,1.9)	0	NA	NA	0	NA	NA
ESRD	9	0.2 (0.1,0.3)	2	0.1 (0.0,0.4)	0.7 (0.1,5.1)	0	NA	NA	1	10.0 (0.4,63.4)	62.2 (7.9,490.9)
AKD	12	0.2 (0.1,0.4)	5	0.4 (0.2,0.8)	1.8 (0.6,5.1)	1	0.5 (0.0,3.7)	2.5 (0.3,19.3)	0	NA	NA
All-cause mortality	265	4.5 (4.0,5.1)	50	3.5 (2.6,4.5)	0.8 (0.5,1.0)	9	9.3 (4.8,17.2)	2.0 (1.1,3.9)	4	68.7 (27.9,175.7)	15.1 (5.7,39.9)
**Age (40-65)**											
Major CVD	2,856	42.2 (40.7,43.8)	671	42.8 (39.6,46.1)	1.0 (0.9,1.1)	63	51.0 (39.8,65.1)	1.2 (0.9,1.6)	7	71.3 (30.4,141.0)	1.7 (0.8,3.5)
Heart failure	286	4.1 (3.6,4.6)	78	4.8 (3.8,5.9)	1.2 (0.9,1.5)	7	5.1 (2.2,10.1)	1.3 (0.6,2.7)	0	NA	NA
Carditis	40	0.6 (0.4,0.8)	8	0.5 (0.2,0.9)	0.8 (0.4,1.8)	1	1.0 (0.2,4.3)	1.8 (0.4,8.6)	2	16.9 (2.5,56.4)	29.9 (7.5,119.5)
Stroke	1,519	21.9 (20.8,23.0)	326	20.3 (18.2,22.6)	0.9 (0.8,1.1)	29	23.2 (16.0,32.8)	1.1 (0.7,1.5)	3	30.6 (6.5,75.5)	1.4 (0.5,3.8)
AF	269	3.8 (3.4,4.3)	85	5.2 (4.2,6.4)	1.4 (1.1,1.8)	6	4.5 (1.7,9.0)	1.2 (0.5,2.6)	0	NA	NA
Flutter	44	0.6 (0.5,0.8)	14	0.9 (0.5,1.4)	1.4 (0.7,2.7)	0	NA	NA	0	NA	NA
CHD	1,270	18.4 (17.4,19.4)	319	19.9 (17.8,22.2)	1.1 (1.0,1.2)	33	26.4 (18.6,36.5)	1.4 (1.0,2.1)	4	40.3 (11.3,91.1)	2.2 (0.8,5.9)
ACS	1,018	14.5 (13.6,15.4)	228	14.0 (12.3,15.9)	1.0 (0.8,1.1)	24	18.3 (12.0,26.8)	1.3 (0.8,1.9)	3	27.7 (6.3,74.1)	1.9 (0.6,5.8)
DVT	111	1.6 (1.3,1.9)	24	1.4 (0.9,2.1)	0.9 (0.6,1.5)	3	2.6 (0.8,6.8)	1.7 (0.5,5.4)	0	NA	NA
COPD	226	3.2 (2.8,3.7)	74	4.6 (3.6,5.7)	1.4 (1.1,1.9)	5	3.7 (1.3,8.0)	1.2 (0.4,3.1)	0	NA	NA
SLD	91	1.3 (1.0,1.6)	22	1.4 (0.9,2.0)	1.1 (0.6,1.7)	3	2.1 (0.5,5.6)	1.6 (0.6,4.3)	0	NA	NA
Pancreatitis	140	2.0 (1.7,2.3)	31	1.9 (1.3,2.6)	0.9 (0.6,1.4)	2	1.6 (0.5,5.6)	0.8 (0.2,3.5)	1	11.8 (2.5,57.7)	5.9 (0.8,42.3)
ESRD	37	0.5 (0.4,0.7)	8	0.5 (0.2,0.9)	0.9 (0.4,2.0)	3	2.2 (0.5,5.6)	3.9 (1.1,13.4)	0	NA	NA
AKD	115	1.6 (1.3,1.9)	33	2.0 (1.4,2.8)	1.3 (0.8,1.9)	1	1.1 (0.2,4.3)	0.7 (0.2,2.8)	1	7.5 (0.3,37.6)	4.6 (0.8,26.0)
All-cause mortality	2,635	37.2 (35.8,38.7)	687	41.9 (38.8,45.1)	1.1 (1.0,1.2)	147	113.3 (96.4,133.1)	3.1 (2.6,3.6)	37	370.2 (262.9,503.0)	9.9 (7.0,14.0)
**Age≥65**											
Major CVD	5,564	131.2 (127.8,134.7)	1,223	156.5 (147.9,165.4)	1.2 (1.1,1.3)	229	204.4 (179.5,232.5)	1.6 (1.4,1.8)	44	183.9 (137.2,246.8)	1.4 (1.0,1.9)
Heart failure	1,545	22.4 (21.3,23.6)	439	34.3 (31.2,37.7)	1.5 (1.4,1.7)	99	53.7 (43.8,64.9)	2.4 (2.0,2.9)	23	55.7 (35.9,82.1)	2.4 (1.7,3.4)
Carditis	71	1.4 (1.1,1.7)	16	1.6 (0.9,2.6)	1.2 (0.7,2.1)	2	1.6 (0.4,5.2)	1.2 (0.4,4.2)	0	NA	NA
Stroke	3,290	51.2 (49.5,53.0)	683	57.3 (53.1,61.7)	1.1 (1.0,1.2)	110	64.6 (53.2,77.3)	1.3 (1.0,1.5)	19	51.5 (33.1,80.4)	1.0 (0.6,1.5)
AF	1,086	21.8 (20.6,23.2)	281	30.5 (27.0,34.2)	1.4 (1.2,1.6)	50	37.9 (28.5,49.5)	1.7 (1.3,2.4)	8	26.7 (12.0,50.1)	1.2 (0.5,3.0)
Flutter	89	1.7 (1.4,2.1)	28	3.0 (2.0,4.3)	1.7 (1.1,2.6)	1	1.0 (0.2,4.1)	0.6 (0.2,2.0)	1	1.7 (0.1,12.3)	1.0 (0.2,4.0)
CHD	2,194	34.0 (32.6,35.5)	504	41.9 (38.4,45.7)	1.2 (1.1,1.4)	94	54.0 (43.8,65.7)	1.6 (1.3,2.0)	20	50.7 (31.5,76.6)	1.5 (0.9,2.4)
ACS	1,840	36.3 (34.6,38.0)	417	44.2 (40.1,48.6)	1.2 (1.1,1.4)	72	53.1 (42.1,66.7)	1.5 (1.2,1.8)	13	44.5 (25.8,75.0)	1.2 (0.7,2.1)
DVT	175	3.4 (2.9,3.9)	55	5.7 (4.3,7.3)	1.7 (1.2,2.3)	11	7.8 (3.9,13.2)	2.3 (1.4,3.9)	8	25.5 (11.4,47.5)	7.5 (2.6,21.7)
COPD	556	11.1 (10.2,12.1)	146	15.8 (13.3,18.5)	1.4 (1.2,1.7)	33	25.0 (17.8,35.0)	2.3 (1.6,3.2)	6	20.0 (7.7,40.8)	1.8 (0.8,4.1)
SLD	114	2.2 (1.8,2.6)	23	2.4 (1.5,3.4)	1.1 (0.7,1.7)	3	1.9 (0.4,5.2)	0.9 (0.4,2.0)	3	9.0 (2.0,23.6)	4.1 (0.9,18.1)
Pancreatitis	236	4.5 (4.0,5.2)	58	6.0 (4.7,7.8)	1.3 (1.0,1.8)	10	7.1 (3.4,12.3)	1.6 (0.8,3.0)	3	9.3 (2.0,23.9)	2.0 (0.9,4.7)
ESRD	99	1.9 (1.5,2.3)	23	2.4 (1.6,3.6)	1.3 (0.8,2.0)	8	5.7 (2.9,11.3)	3.0 (1.5,6.0)	1	2.5 (0.1,12.1)	1.3 (0.4,4.6)
AKD	537	10.4 (9.6,11.3)	119	12.4 (10.3,14.8)	1.2 (1.0,1.5)	29	21.3 (14.7,30.2)	2.1 (1.5,2.8)	12	40.3 (20.9,66.4)	3.9 (2.1,7.3)
All-cause mortality	14,282	274.6 (270.1,279.1)	4,045	417.8 (405.1,430.8)	1.5 (1.5,1.6)	1,191	850.7 (803.2,899.8)	3.1 (2.9,3.3)	493	1610.4 (1474.3,1758.8)	5.8 (5.3,6.5)

Note: Major CVD: composite outcomes of heart failure, stroke and coronary artery disease; AF: Atrial fibrillation; CHD: coronary heart disease ACS: Acute coronary disorder; DVT: Deep vein thrombosis; COPD: Chronic pulmonary disease; SLD: Severe liver disease; ESRD: End stage renal disease; AKD: Acute kidney disease; NA: Not available due to insufficient number

Hazard Ratio was obtained by Cox regression adjusted with weighting

In those aged under 65, the critically exposed group was affected by COVID-19 infection severely and was more likely to develop the cardiovascular outcome of carditis (HR (aged ≤ 40): 17.0 (95%CI: 2.4,122.8); HR (40 < aged < 65): 29.9 (95%CI: 7.5,119.5)), and all-cause mortality (HR (aged ≤ 40): 15.1 (95%CI: 5.7,39.9); HR (40 < aged < 65): 9.9 (95%CI: 7.0,14.0)).

In the elderly cohort (age ≥ 65), irrespective of the degree of severity, patients with COVID-19 infection were associated with significantly higher risks of most of the outcomes compared to the unexposed group. Particularly, increasing risks were observed with higher initial disease severity, including heart failure (HR (mild–moderate): 1.5 (95%CI: 1.4,1.7), HR (severe): 2.4 (95%CI: 2.0,2.9), HR (critical): 2.4 (95%CI: 1.7,3.4)), DVT (HR (mild): 1.7 (95%CI: 1.2,2.3), HR (severe): 2.3 (95%CI: 1.4,3.9), HR (critical): 7.5 (95%CI: 2.6,21.7)), and all-cause mortality (HR (mild): 1.5 (95%CI: 1.5,1.6), HR (severe): 3.1 (95%CI: 2.9,3.3), HR (critical): 5.8 (95%CI: 5.3,6.5)).

The subgroup analyses showed a higher increase in risks associated with COVID-19 sequelae among female patients. Further, fully vaccinated patients were observed to have a lower risk of sequelae compared to those patients with less than two doses of vaccination (Supplementary 5–12).

After conducting Bonferroni correction, using Bonferroni-corrected *α* as 0.001 and based on the *p*-value from Supplementary tables 14 and 15, the family-wise error rate is reduced from 10% to 4.4%. Almost all the false hypotheses continue to be rejected significantly based on the Bonferroni-corrected *α* and the increased risk of multiple different disease outcomes maintained in both the acute and post-acute phases, with increasing severity of initial COVID-19 illness, especially among the elderly.

## Discussion

Previously, in addition to respiratory complications, clinical and longitudinal studies have identified complications in COVID-19 patients post infection (acute phase), including cardiovascular, neurological, gastrointestinal, renal, and hepatic disease conditions [37, 38]. Over time, these symptoms may persist and develop into a variety of multi-organ sequelae in the long-term (post-acute phase). Hence, a comprehensive list of such probable disease outcomes affecting different organs was identified and analyzed spanning both phases of infection in the same cohort of patients, stratified by initial disease severity using clinical parameters like viral load and hospitalization/oxygen support records, as well as the biomarkers—CRP and D-dimer—in this study. Overall, the findings from this study reveal a significant increase in incident risks associated with several disease complications in infected patients (exposed group), particularly those identified with severe and critical initial severity of infection and aged > 40, compared to those without COVID-19 (potentially contributing to the substantially higher risks of all-cause mortality—found to be associated with infected patients in both phases of infection). A general trend tending towards a progressive increase in these risks in an exposure–response relationship manner with increasing initial disease severity (from mild–moderate to severe and critical) and advancing age (from age ≤ 40 to between 40–65 and > 65) was also identified. However, the findings in that older and severer patients were associated with higher risks of several diseases’ complications might also apply to other respiratory infections other than COVID-19. Notably, patients identified with “mild–moderate” disease severity based on the selection criteria, were associated with either insignificant risks (observed in patients aged ≤ 40) or the lowest risks (in those aged > 40) for the disease outcomes in the acute phase of infection; while in the post-acute phase, these risks were largely insignificant in those aged < 65, remaining significant only in the elderly (age ≥ 65). In accordance to a previous study on the persistence in the risk of PASC between patients of different vaccination statuses [39], fully vaccinated patients were observed to have a lower risk of sequelae compared to those patients with less than two doses of vaccination overall.

The findings from this study are strengthened by being consistent with prior findings. Initially, several independent clinical studies observed elevated levels of D-dimer and CRP (independently as well as in combination with other biomarkers) in hospitalized and/or critically ill COVID-19 patients linked with hyperinflammation [40]; later found to be associated with increased hospitalization risk (linked to the severity of infection) and/or mortality risk in such patients. While evidence of a direct relationship of viral load with severe COVID-19 infection is inconsistent, previous studies reported higher viral loads to be associated with older age and increased mortality risk [41]. Given that advancing age is also well recognized as a risk factor for severe COVID-19 infection, poor prognosis, and is associated with the development of multi-organ sequelae post infection, a narrative review hypothesized the possibility of a mechanistic relationship linking increased COVID-19 severity and mortality risk with higher viral loads based on clinical studies [41]. Another review highlighted higher viral load as an independent predictor of disease severity and its association with higher levels of inflammatory immune biomarkers including CRP and D-dimer based on immunological studies [42]. Further, some studies found that patient groups with higher prevalence of severe COVID-19 infection demonstrated elevated D-dimer and CRP levels. Indeed, a meta-analysis evaluating a pool of 15,000 patients identified from 29 independent clinical studies demonstrated that diabetic COVID-19 patients showed higher levels of CRP (SMD 0.41 mg/L, 95% CI (0.21–0.60) mg/L) and concentration of D-Dimer (SMD 0.32 mg/L, 95% CI (0.17–0.47) mg/L) than the non-diabetic patients [43]. In addition, higher serum CRP and D-Dimer levels were correlated positively with increasing age of COVID-19 patients by the same study. Since diabetes is also associated with increasing risk of COVID-19-associated disease outcomes and mortality [44], altogether, these findings substantiate the overall conclusion drawn from this study that COVID-19 patient groups prone to more severe infection are more likely to develop disease outcomes/mortality and can be identified by elevated levels of CRP and D-dimer and higher viral loads to be classified into a high-risk patient group. In fact, a recent study reported CRP to be the best at discriminating between severe and non-severe COVID-19 compared to various other hyperinflammatory biomarkers [45]. The same findings held true even in an outpatient setting and in the post-acute phase of infection, illustrated by an example of a cross-sectional study of ~ 400 COVID-19-diagnosed patients treated as outpatients reporting elevated levels of D-dimer in 15% of participants (tending to be older in age) even after a median of 255 days post initial infection [46]. Based on a multivariable logistic regression analysis, this cross-sectional study also identified a significant positive association between the CRP and D-dimer levels (13% of patients with elevated D-dimer presented concomitantly with abnormal CRP levels), supporting the usage of these two clinical parameters to stratify COVID-19 infected patients in the present study. Indeed, a few prior studies have attempted to use clinical parameters to predict the risk of disease progression and evaluated the risks associated with the development of certain outcomes. A multi-center cohort study enrolling 239 COVID-19 patients observed disease progression in ~ 40% of the patients in the hospital, identifying CRP levels > median value of (OR, 2.25; 95% C.I., 1.02–4.99) as an independent risk factor for progression [47]. These patients were found to be associated with higher risks of developing acute kidney injury, cardiac injury, respiratory failure, ARDS, and death, in addition to facing longer hospital stay and reduction in viral load (conversion to CT value < 30), compared to those without disease progression [47]. Another single-center, retrospective, cohort review study of 100 hospitalized patients identified elevated D-dimer at different cutoff values to be associated with predicting in-hospital mortality and risk of developing acute kidney injury in infected patients [48].

While the exact pathophysiology and manifestation of COVID-19 in both phases of infection is not completely understood, several lines of probable mechanism have been proposed and summarized by previous narrative reviews [4, 5]. Briefly, viral-induced organ damage mediated via initial viral entry through the interaction of SARS-CoV-2 with the angiotensin-converting enzyme 2 (ACE2) receptor found to be highly expressed in the lungs, heart, gastrointestinal tract, central/nervous system, and pancreas [49–51] is believed to be a major mechanism for leading to dysfunction of multiple different organs during the acute phase of infection. Whereas, for the post-acute phase, viral persistence due to the formation of “reservoirs” of viral remnants and the virus itself in ACE-2-expressing organs (including those already infected during the initial acute phase of the disease), leading to prolonged inflammation inciting an abnormal immunological response of excessive cytokines and pro-inflammatory molecules secretion is believed to be one of the leading mechanisms attributed to the development of COVID-19-associated disease outcomes. Further, the development of microthrombi in the systemic vasculature enhancing abnormal pro-coagulation in the acute phase [52, 53] may contribute to thrombotic complications such as stroke and DVT and also lead to cerebral infarctions affecting the blood–brain barrier and oxygen supply, possibly resulting in seizures, as observed in this study [54]. Altogether, this may explain why clinical biomarkers such as CRP and D-dimer are good predictors of COVID-19-associated disease progression and complications, since these proteins are rapidly secreted in response to infections within hours, as part of establishing a systemic inflammatory state to trigger host immune response [55, 56]. Although the exact underlying pathophysiological mechanisms linking these biomarkers specifically to manifestation of severe COVID-19 and associated mortality remain poorly understood, it is known that elevated levels of CRP (synthesized by the liver and mediated by the cytokine interleukin 6 (IL-6)) are commonly triggered by severe infections, tissue/organ injury, autoimmune conditions, and cardiovascular events (especially pericarditis) [57]; while elevated levels of D-dimer (present in the blood as a small protein fragment of fibrin degraded during dissolving of a thrombus) reflect active coagulation, vascular endothelial damage, and/or fibrinolytic processes [58, 59].

There are several strengths underlying strengths in this study. Firstly, a short- and long-term follow-up in the same patient cohort allowed monitoring of the development of an extensive list of multiple pre-specified different disease outcomes over 28 months, and the dynamic changes in the associated risks, giving an insight into the manifestation of COVID-19 infection over time. Secondly, incorporating the use of established clinical parameters including biomarkers and viral load to define the levels of severity of infection (rather than relying on hospitalization/oxygen support records as a proxy subject to biases in hospital bed/treatment availability) increases the accuracy of the findings and provides a biological context for interpreting the results. Nevertheless, some limitations still remain. Firstly, being an observational study, only the association between COVID-19 infection and risks for the specific disease outcomes can be established, rather than causality. Secondly, owing to the limited sample size of the severely and critically COVID-19 exposed group, especially younger patients, further analysis in future studies is warranted. Thirdly, since the exposed group was distinguished from the unexposed group based on the latter not having a positive COVID-19 PCR test result and/or not being hospitalized with a COVID-19-related diagnosis admission code, the possibility of asymptomatic, undiagnosed COVID-19-infected individuals being included in the control group and/or excluded from recruitment into the COVID-19 patient cohort still remains; however, this should only bias the results towards the null. Besides, although the severity criteria were carefully deliberated based on a literature review, using the single indicator might introduce bias caused by misclassification. Hence, further study is needed for precisely identifying the severity criteria. Moreover, indication or detection bias might arise, leading to the possible under-reporting of existing conditions before receiving a diagnosis of COVID-19. Lastly, risks of certain complications may not have reached statistical significance stemming from the inherent rarity of the outcome and consequent low prevalence in COVID-19 patients, resulting in low event rates and high confidence intervals.

## Conclusions

The risk of disease outcomes increased significantly in an exposure–response relationship manner with increasing severity of acute COVID-19 illness in both acute and post-acute phases. Future studies are warranted to validate these findings by considering more clinical parameters to stratify COVID-19 patients by initial disease severity and analyzing risks associated with COVID-19-related disease outcomes in other populations with larger cohorts. In addition, future studies may benefit from the risk stratification of patients based on clearly defined and universally accepted cutoff threshold levels for clinical parameters especially biomarkers based on biological evidence from immunological studies.

## Supplementary Information

Supplementary Material 1.

## Data Availability

Data underlying in this study cannot be shared publicly due to the privacy of individuals.

## Abbreviations

PASC: Post-acute sequelae SARS-CoV-2 infection
CRP: C-reactive protein
CT: Cycle threshold
PCR: Polymerase chain reaction
HA: Hong Kong Hospital Authority
DH: Department of Health
RAT: Rapid antigen test
ICD-9-CM: International Classification of Diseases, Ninth Revision, Clinical Modification
Major CVD: Major cardiovascular disease
CHD: Coronary heart disease
ACS: Acute coronary syndrome
DVT: Deep vein thrombosis
COPD: Chronic obstructive pulmonary diseases
ESRD: End-stage renal disease
AKD: Acute kidney disease
CCI: Charlson’s comorbidity index
CIs: Confidence intervals
SMD: Standardized mean difference
HRs: Hazard ratios
ACE2: Angiotensin-converting enzyme 2
IL-6: Interleukin 6
