# Supplementary material for: Association of COVID-19 with acute and post-acute risk of multiple different complications and mortality in patients infected with omicron variant stratified by initial disease severity: a cohort study in Hong Kong
Source: BMC Med. 2024 Oct 14;22:461. doi: 10.1186/s12916-024-03630-6 (PMC11476291; doi:10.1186/s12916-024-03630-6)
Supplement: Supplementary file 1 — Supplementary Material 1. [file 12916_2024_3630_MOESM1_ESM.docx]

**Supplementary table 1. Disease definition**

| Disease | ICD-9-CM |
| --- | --- |
| Myocardial infarction | 410 |
| Heart failure | 428,398.91,402.01,402.11,402.91,404.01,404.03, 404.11,404.13,404.91,404.93 |
| Carditis | 420.9, 423.9, 422.90 |
| Stroke | 430-438 |
| Atrial fibrillation | 427.3 |
| Arrythmias, atrial flutter | 427.32 |
| coronary heart disease | 410-414, 36.0, 36.1, V45.81 |
| Acute coronary disease | 410, 411, 36.0, 36.1, V45.81 |
| Deep vein thrombosis | 453 |
| Chronic pulmonary disease and allied conditions | 490-496 |
| Sever liver disease | 456.0-456.2, 572.2, 572.3, 572.4, 572.8 |
| Pancreatitis | 577.0, 577.1 |
| End stage renal disease | 585.6, 586 |
| Acute kidney disease | 584.5-584.9 |
| Cardiovascular disease | 398.91, 401-405, 410-412, 425.4, 425.5, 425.7-425.9, 427.3, 428, 433, 434, 436-438, 453.8 |
| **Charlson’s Comorbidity** | |
| Myocardial infarction | 410 |
| Dementia | 290 |
| Congestive heart failure | 398.91, 402.01, 402.11, 402.91, 404.01, 404.03, 404.11, 404.13, 404.91, 404.93, 428 |
| Peripheral vascular disease | 441, 443.9, 785.4 |
| Cerebrovascular disease | 430-438 |
| Rheumatoid arthritis and other inflammatory polyarthropathies | 710.0, 710.1, 710.4, 714.0, 714.1, 714.2, 714.81, 725 |
| Chronic pulmonary disease | 490-496, 500-505, 506.4 |
| Hemiplegia or paraplegia | 342, 344.1 |
| Chronic renal failure | 582, 585, 586, 588, 583.0-583.2, 583.4, 583.6, 583.7 |
| Liver disease, mild | 571.2, 571.4-571.6 |
| Diabetes without chronic complications | 250.0-250.3, 250.7 |
| Diabetes with chronic complications | 250.4-250.6 |
| Ulcers | 531-534 |
| Metastatic solid tumor | 196-199 |
| Any malignancy | 140-149, 150-159, 180-189, 170-172, 174, 175, 176, 179, 160-165, 190-195, 200-208 |
| Liver disease, moderate to severe | 456.0-456.2, 572.2-572.4. 572.8 |
| AIDS | 042 |
| Charlson’s Comorbidity Index Formula | Age score+ Myocardial infarction+ Congestive heart failure+ Peripheral vascular disease+ Cerebrovascular disease+ Chronic pulmonary disease+ Dementia+ Hemiplegia or paraplegia*2+ Diabetes without chronic complications+ Diabetes with chronic complications*2+ Chronic renal failure*2+ Liver disease, mild+ Liver disease, moderate to severe*3+ Ulcers+ Rheumatoid arthritis and other inflammatory polyarthropathies+ AIDS*6+ Metastatic solid tumour*2+ Any malignancy*6 |

**Supplementary table 2. Procedure definition in HK dataset (ICD-9)**

| ICD-9-codes (HK) | Procedure |
| --- | --- |
| 39.65 | Extracorporeal Membrane Oxygenation (Ecmo) |
| 89.18 | Other Sleep Disorder Function Tests |
| 93.90 | Non-Invasive Mechanical Ventilation |
| 93.95 | Hyperbaric Oxygenation |
| 93.96 | Other Oxygen Enrichment |
| 96.7x | Other Continuous Invasive Mechanical Ventilation |
| 96.04 | Insertion of Endotracheal Tube |

Supplementary Table 3 Baseline characteristics of patients before weighting (Acute phase)

| **Characteristics** | **Control** | **Mild** | **Severity** | **Critical** | **SMD** |
| --- | --- | --- | --- | --- | --- |
| **Age ≤40** | **N=1,143,510** | **N=286,114** | **N=18,419** | **N=1,168** |  |
| Age, years (mean (SD)) | 25.13 (10.54) | 26.23 (10.34) | 25.73 (11.05) | 23.48 (12.25) | 0.13 |
| Sex, male (%) | 540145 (47.2) | 134916 (47.2) | 8593 (46.7) | 575 (49.2) | 0.03 |
| Charlson Comorbidity Index (mean (SD)) | 0.03 (0.26) | 0.04 (0.27) | 0.06 (0.41) | 0.22 (0.86) | 0.16 |
| Renin-angiotensin-system agents (%) | 7096 (0.6) | 2392 (0.8) | 219 (1.2) | 35 (3.0) | 0.10 |
| Beta blockers (%) | 7498 (0.7) | 2242 (0.8) | 239 (1.3) | 42 (3.6) | 0.11 |
| Calcium channel blockers (%) | 7976 (0.7) | 2644 (0.9) | 251 (1.4) | 41 (3.5) | 0.11 |
| Diuretics (%) | 1352 (0.1) | 409 (0.1) | 72 (0.4) | 17 (1.5) | 0.09 |
| Nitrates (%) | 329 (0.0) | 110 (0.0) | 10 (0.1) | 7 (0.6) | 0.05 |
| Lipid lowering agents (%) | 5929 (0.5) | 1951 (0.7) | 163 (0.9) | 25 (2.1) | 0.08 |
| Insulins (%) | 2252 (0.2) | 632 (0.2) | 70 (0.4) | 17 (1.5) | 0.08 |
| Antidiabetic drugs (%) | 5424 (0.5) | 1787 (0.6) | 129 (0.7) | 25 (2.1) | 0.08 |
| Oral anticoagulants (%) | 517 (0.0) | 137 (0.0) | 15 (0.1) | 9 (0.8) | 0.06 |
| Antiplatelets (%) | 2133 (0.2) | 692 (0.2) | 80 (0.4) | 14 (1.2) | 0.07 |
| Immunosuppressants (%) | 2953 (0.3) | 858 (0.3) | 95 (0.5) | 10 (0.9) | 0.05 |
| Dose status (%) |  |  |  |  | 0.25 |
| 0 | 243022 (21.3) | 43749 (15.3) | 3710 (20.1) | 326 (27.9) |  |
| 1 | 132681 (11.6) | 34715 (12.1) | 1870 (10.2) | 198 (17.0) |  |
| 2 | 492610 (43.1) | 134591 (47.0) | 9443 (51.3) | 444 (38.0) |  |
| 3 | 275197 (24.1) | 73059 (25.5) | 3396 (18.4) | 200 (17.1) |  |
| **40<Age<65** | **N=1,369,365** | **N=320,304** | **N=23,678** | **N=2,261** |  |
| Age, years (mean (SD)) | 53.62 (6.92) | 53.36 (6.98) | 53.67 (6.85) | 55.42 (6.82) | 0.15 |
| Sex, male (%) | 576117 (42.1) | 134436 (42.0) | 9522 (40.2) | 1354 (59.9) | 0.20 |
| Charlson Comorbidity Index (mean (SD)) | 1.19 (1.13) | 1.17 (1.14) | 1.42 (1.57) | 2.40 (2.24) | 0.38 |
| Renin-angiotensin-system agents (%) | 153026 (11.2) | 40100 (12.5) | 3316 (14.0) | 566 (25.0) | 0.19 |
| Beta blockers (%) | 91270 (6.7) | 23416 (7.3) | 2267 (9.6) | 477 (21.1) | 0.23 |
| Calcium channel blockers (%) | 209839 (15.3) | 55209 (17.2) | 4464 (18.9) | 652 (28.8) | 0.17 |
| Diuretics (%) | 22045 (1.6) | 5813 (1.8) | 897 (3.8) | 291 (12.9) | 0.25 |
| Nitrates (%) | 15876 (1.2) | 3995 (1.2) | 484 (2.0) | 92 (4.1) | 0.10 |
| Lipid lowering agents (%) | 211489 (15.4) | 53905 (16.8) | 4419 (18.7) | 680 (30.1) | 0.19 |
| Insulins (%) | 17334 (1.3) | 4424 (1.4) | 614 (2.6) | 197 (8.7) | 0.19 |
| Antidiabetic drugs (%) | 120508 (8.8) | 32345 (10.1) | 2514 (10.6) | 472 (20.9) | 0.18 |
| Oral anticoagulants (%) | 5260 (0.4) | 1376 (0.4) | 232 (1.0) | 67 (3.0) | 0.12 |
| Antiplatelets (%) | 62169 (4.5) | 15511 (4.8) | 1545 (6.5) | 346 (15.3) | 0.20 |
| Immunosuppressants (%) | 7189 (0.5) | 1820 (0.6) | 304 (1.3) | 52 (2.3) | 0.09 |
| Dose status (%) |  |  |  |  | 0.38 |
| 0 | 158472 (11.6) | 19328 (6.0) | 2328 (9.8) | 544 (24.1) |  |
| 1 | 86759 (6.3) | 23440 (7.3) | 1600 (6.8) | 314 (13.9) |  |
| 2 | 520286 (38.0) | 136556 (42.6) | 11580 (48.9) | 830 (36.7) |  |
| 3 | 603848 (44.1) | 140980 (44.0) | 8170 (34.5) | 573 (25.3) |  |
| **Age≥65** | **N=1,012,177** | **N=194,227** | **N=31,505** | **N=10,178** |  |
| Age, years (mean (SD)) | 74.72 (7.95) | 74.26 (8.03) | 79.57 (9.54) | 82.28 (9.26) | 0.55 |
| Sex, male (%) | 463627 (45.8) | 93291 (48.0) | 16856 (53.5) | 6135 (60.3) | 0.16 |
| Charlson Comorbidity Index (mean (SD)) | 3.59 (1.48) | 3.62 (1.57) | 4.66 (1.97) | 5.16 (1.98) | 0.54 |
| Renin-angiotensin-system agents (%) | 289836 (28.6) | 61226 (31.5) | 11294 (35.8) | 3570 (35.1) | 0.09 |
| Beta blockers (%) | 185963 (18.4) | 39432 (20.3) | 7971 (25.3) | 2640 (25.9) | 0.11 |
| Calcium channel blockers (%) | 418382 (41.3) | 87427 (45.0) | 15267 (48.5) | 5059 (49.7) | 0.10 |
| Diuretics (%) | 70053 (6.9) | 15834 (8.2) | 5426 (17.2) | 2048 (20.1) | 0.24 |
| Nitrates (%) | 52479 (5.2) | 12027 (6.2) | 3297 (10.5) | 1104 (10.8) | 0.13 |
| Lipid lowering agents (%) | 434916 (43.0) | 90643 (46.7) | 15651 (49.7) | 4816 (47.3) | 0.07 |
| Insulins (%) | 33024 (3.3) | 7532 (3.9) | 2434 (7.7) | 999 (9.8) | 0.16 |
| Antidiabetic drugs (%) | 215864 (21.3) | 46964 (24.2) | 8166 (25.9) | 2711 (26.6) | 0.07 |
| Oral anticoagulants (%) | 31726 (3.1) | 6976 (3.6) | 2261 (7.2) | 789 (7.8) | 0.13 |
| Antiplatelets (%) | 186834 (18.5) | 40179 (20.7) | 10116 (32.1) | 3757 (36.9) | 0.25 |
| Immunosuppressants (%) | 4818 (0.5) | 1147 (0.6) | 343 (1.1) | 142 (1.4) | 0.06 |
| Dose status (%) |  |  |  |  | 0.53 |
| 0 | 206511 (20.4) | 30856 (15.9) | 11045 (35.1) | 4785 (47.0) |  |
| 1 | 126483 (12.5) | 30817 (15.9) | 5749 (18.2) | 2273 (22.3) |  |
| 2 | 344628 (34.0) | 71407 (36.8) | 8971 (28.5) | 2212 (21.7) |  |
| 3 | 334555 (33.1) | 61147 (31.5) | 5740 (18.2) | 908 (8.9) |  |
|  |  |  |  |  |  |

Supplementary Table 4. Baseline characteristics of patients before weighting (Post-acute phase)

| **Characteristics** | **Control** | **Mild** | **Severity** | **Critical** | **SMD** |
| --- | --- | --- | --- | --- | --- |
| **Age ≤40** | **N=1,143,464** | **N=286,103** | **N=18,396** | **N=1,148** |  |
| Age, years (mean (SD)) | 25.13 (10.54) | 26.23 (10.34) | 25.72 (11.05) | 23.43 (12.25) | 0.13 |
| Sex, male (%) | 540119 (47.2) | 134911 (47.2) | 8578 (46.6) | 563 (49.0) | 0.02 |
| Charlson Comorbidity Index (mean (SD)) | 0.03 (0.26) | 0.04 (0.27) | 0.06 (0.39) | 0.21 (0.85) | 0.16 |
| Renin-angiotensin-system agents (%) | 7095 (0.6) | 2392 (0.8) | 214 (1.2) | 34 (3.0) | 0.10 |
| Beta blockers (%) | 7491 (0.7) | 2241 (0.8) | 233 (1.3) | 38 (3.3) | 0.11 |
| Calcium channel blockers (%) | 7975 (0.7) | 2644 (0.9) | 246 (1.3) | 40 (3.5) | 0.11 |
| Diuretics (%) | 1342 (0.1) | 407 (0.1) | 68 (0.4) | 16 (1.4) | 0.08 |
| Nitrates (%) | 329 (0.0) | 110 (0.0) | 9 (0.0) | 6 (0.5) | 0.05 |
| Lipid lowering agents (%) | 5927 (0.5) | 1950 (0.7) | 162 (0.9) | 24 (2.1) | 0.08 |
| Insulins (%) | 2249 (0.2) | 631 (0.2) | 67 (0.4) | 15 (1.3) | 0.07 |
| Antidiabetic drugs (%) | 5422 (0.5) | 1787 (0.6) | 127 (0.7) | 22 (1.9) | 0.07 |
| Oral anticoagulants (%) | 517 (0.0) | 137 (0.0) | 15 (0.1) | 8 (0.7) | 0.06 |
| Antiplatelets (%) | 2132 (0.2) | 692 (0.2) | 77 (0.4) | 12 (1.0) | 0.06 |
| Immunosuppressants (%) | 2952 (0.3) | 858 (0.3) | 95 (0.5) | 10 (0.9) | 0.05 |
| Dose status (%) |  |  |  |  | 0.25 |
| 0 | 243001 (21.3) | 43744 (15.3) | 3695 (20.1) | 317 (27.6) |  |
| 1 | 132674 (11.6) | 34714 (12.1) | 1867 (10.1) | 195 (17.0) |  |
| 2 | 492598 (43.1) | 134589 (47.0) | 9439 (51.3) | 438 (38.2) |  |
| 3 | 275191 (24.1) | 73056 (25.5) | 3395 (18.5) | 198 (17.2) |  |
| **40<Age<65** | **N=1,368,889** | **N=320,126** | **N=23,490** | **N=2,011** |  |
| Age, years (mean (SD)) | 53.62 (6.92) | 53.36 (6.98) | 53.63 (6.84) | 55.13 (6.91) | 0.13 |
| Sex, male (%) | 575817 (42.1) | 134326 (42.0) | 9402 (40.0) | 1189 (59.1) | 0.19 |
| Charlson Comorbidity Index (mean (SD)) | 1.19 (1.12) | 1.17 (1.14) | 1.40 (1.52) | 2.21 (2.05) | 0.34 |
| Renin-angiotensin-system agents (%) | 152943 (11.2) | 40061 (12.5) | 3273 (13.9) | 502 (25.0) | 0.19 |
| Beta blockers (%) | 91175 (6.7) | 23375 (7.3) | 2214 (9.4) | 403 (20.0) | 0.21 |
| Calcium channel blockers (%) | 209721 (15.3) | 55161 (17.2) | 4407 (18.8) | 564 (28.0) | 0.16 |
| Diuretics (%) | 21901 (1.6) | 5776 (1.8) | 830 (3.5) | 227 (11.3) | 0.22 |
| Nitrates (%) | 15848 (1.2) | 3988 (1.2) | 474 (2.0) | 82 (4.1) | 0.10 |
| Lipid lowering agents (%) | 211395 (15.4) | 53865 (16.8) | 4365 (18.6) | 599 (29.8) | 0.18 |
| Insulins (%) | 17268 (1.3) | 4404 (1.4) | 584 (2.5) | 148 (7.4) | 0.17 |
| Antidiabetic drugs (%) | 120420 (8.8) | 32309 (10.1) | 2478 (10.5) | 399 (19.8) | 0.16 |
| Oral anticoagulants (%) | 5233 (0.4) | 1368 (0.4) | 224 (1.0) | 58 (2.9) | 0.11 |
| Antiplatelets (%) | 62104 (4.5) | 15481 (4.8) | 1505 (6.4) | 293 (14.6) | 0.19 |
| Immunosuppressants (%) | 7177 (0.5) | 1817 (0.6) | 292 (1.2) | 44 (2.2) | 0.09 |
| Dose status (%) |  |  |  |  | 0.35 |
| 0 | 158249 (11.6) | 19237 (6.0) | 2217 (9.4) | 425 (21.1) |  |
| 1 | 86709 (6.3) | 23412 (7.3) | 1570 (6.7) | 271 (13.5) |  |
| 2 | 520156 (38.0) | 136512 (42.6) | 11539 (49.1) | 753 (37.4) |  |
| 3 | 603775 (44.1) | 140965 (44.0) | 8164 (34.8) | 562 (27.9) |  |
| **Age≥65** | **N=1,009,103** | **N=191,678** | **N=27,903** | **N=6,617** |  |
| Age, years (mean (SD)) | 74.69 (7.93) | 74.10 (7.89) | 78.66 (9.27) | 80.68 (9.20) | 0.46 |
| Sex, male (%) | 462005 (45.8) | 91959 (48.0) | 14821 (53.1) | 3909 (59.1) | 0.15 |
| Charlson Comorbidity Index (mean (SD)) | 3.59 (1.47) | 3.60 (1.55) | 4.51 (1.90) | 4.95 (1.94) | 0.48 |
| Renin-angiotensin-system agents (%) | 288740 (28.6) | 60324 (31.5) | 10057 (36.0) | 2407 (36.4) | 0.10 |
| Beta blockers (%) | 185072 (18.3) | 38751 (20.2) | 7001 (25.1) | 1737 (26.3) | 0.12 |
| Calcium channel blockers (%) | 416838 (41.3) | 86188 (45.0) | 13507 (48.4) | 3384 (51.1) | 0.11 |
| Diuretics (%) | 68912.0 (6.8) | 15091.0 (7.9) | 4300.0 (15.4) | 1198.0 (18.1) | 0.21 |
| Nitrates (%) | 52122.0 (5.2) | 11680.0 (6.1) | 2780.0 (10.0) | 705.0 (10.7) | 0.13 |
| Lipid lowering agents (%) | 433580.0 (43.0) | 89512.0 (46.7) | 14067.0 (50.4) | 3293.0 (49.8) | 0.09 |
| Insulins (%) | 32521.0 (3.2) | 7225.0 (3.8) | 1955.0 (7.0) | 603.0 (9.1) | 0.15 |
| Antidiabetic drugs (%) | 215117.0 (21.3) | 46385.0 (24.2) | 7364.0 (26.4) | 1847.0 (27.9) | 0.09 |
| Oral anticoagulants (%) | 31405.0 (3.1) | 6757.0 (3.5) | 1885.0 (6.8) | 505.0 (7.6) | 0.13 |
| Antiplatelets (%) | 185800.0 (18.4) | 39169.0 (20.4) | 8646.0 (31.0) | 2296.0 (34.7) | 0.23 |
| Immunosuppressants (%) | 4779.0 (0.5) | 1119.0 (0.6) | 304.0 (1.1) | 96.0 (1.5) | 0.06 |
| Dose status (%) |  |  |  |  | 0.43 |
| 0 | 204689 (20.3) | 29313 (15.3) | 8693 (31.2) | 2552 (38.6) |  |
| 1 | 126054 (12.5) | 30279 (15.8) | 5070 (18.2) | 1488 (22.5) |  |
| 2 | 344028 (34.1) | 71034 (37.1) | 8479 (30.4) | 1742 (26.3) |  |
| 3 | 334332 (33.1) | 61052 (31.9) | 5661 (20.3) | 835 (12.6) |  |
|  |  |  |  |  |  |

**Supplementary Table 5. Hazard ratio of acute COVID-19 composite outcomes compared to the control groups in female subgroup.**

|  | **Control** | | **Mild** | | | **Severe** | | | **Critical** | | |
| --- | --- | --- | --- | --- | --- | --- | --- | --- | --- | --- | --- |
|  | **Events** | **Incidence rate**  **(per 100,000 person per day)** | **Events** | **Incidence rate**  **(per 100,000 person per day)** | **HR** | **Events** | **Incidence rate**  **(per 100,000 person per day)** | **HR** | **Events** | **Incidence rate**  **(per 100,000 person per day)** | **HR** |
| **Age ≤40** |  |  |  |  |  |  |  |  |  |  |  |
| Major CVD | 4 | 0.02 (0.01,0.06) | 3 | 0.06 (0.01,0.16) | 2.7 (0.7,11.0) | 0 | NA | NA | 1 | 7.39 (1.38,31.78) | 326.5 (58.3,1,827.4) |
| Carditis | 2 | 0.01 (0.00,0.04) | 1 | 0.02 (0.01,0.12) | 2.1 (0.2,22.9) | 1 | 0.45 (0.08,1.89) | 39.4 (3.6,434.8) | 0 | NA | NA |
| AF | 1 | 0.01 (0.00,0.03) | 1 | 0.02 (0.00,0.08) | 3.3 (0.2,53.3) | 0 | NA | NA | 0 | NA | NA |
| Flutter | 0 | NA | 1 | 0.02 (0.00,0.08) | NA | 0 | NA | NA | 0 | NA | NA |
| ACD | 1 | 0.01 (0.00,0.03) | 1 | 0.02 (0.00,0.08) | 3.2 (0.2,50.5) | 0 | NA | NA | 0 | NA | NA |
| DVT | 2 | 0.01 (0.00,0.04) | 1 | 0.02 (0.00,0.08) | 1.6 (0.1,17.9) | 0 | NA | NA | 0 | NA | NA |
| CPD | 3 | 0.02 (0.01,0.05) | 7 | 0.16 (0.06,0.29) | 9.1 (2.3,35.3) | 1 | 0.47 (0.08,1.91) | 27.3 (2.8,262.9) | 0 | NA | NA |
| SLD | 0 | NA | 0 | NA | NA | 0 | NA | NA | 1 | 6.46 (1.37,31.51) | NA |
| Pancreatitis | 2 | 0.01 (0.00,0.04) | 3 | 0.07 (0.02,0.19) | 6.8 (1.2,37.0) | 0 | NA | NA | 0 | NA | NA |
| ESRD | 1 | 0.01 (0.00,0.02) | 0 | NA | NA | 0 | NA | NA | 1 | 6.40 (1.37,31.58) | 1,255.0 (78.9,19,955.7) |
| AKD | 2 | 0.01 (0.00,0.04) | 0 | NA | NA | 0 | NA | NA | 0 | NA | NA |
| All-cause mortality | 20 | 0.11 (0.07,0.16) | 6 | 0.13 (0.06,0.29) | 1.2 (0.5,3.1) | 5 | 1.77 (0.75,3.96) | 16.2 (7.1,36.8) | 4 | 21.67 (6.16,49.57) | 198.5 (79.5,495.5) |
| **Age (40-65)** |  |  |  |  |  |  |  |  |  |  |  |
| Major CVD | 97 | 0.42 (0.34,0.51) | 45 | 0.82 (0.60,1.09) | 2.0 (1.4,2.8) | 9 | 2.30 (1.16,4.14) | 5.5 (2.6,11.8) | 14 | 55.54 (33.24,92.99) | 132.5 (63.2,277.7) |
| Carditis | 5 | 0.02 (0.01,0.04) | 1 | 0.02 (0.00,0.07) | 0.8 (0.1,6.8) | 0 | NA | NA | 0 | NA | NA |
| AF | 14 | 0.06 (0.03,0.09) | 4 | 0.08 (0.03,0.18) | 1.4 (0.4,4.2) | 2 | 0.53 (0.15,1.71) | 9.0 (2.4,34.1) | 0 | NA | NA |
| Flutter | 1 | 0.00 (0.00,0.02) | 0 | NA | NA | 0 | NA | NA | 0 | NA | NA |
| ACD | 15 | 0.06 (0.04,0.10) | 8 | 0.15 (0.07,0.28) | 2.4 (1.0,5.5) | 1 | 0.31 (0.06,1.32) | 4.8 (1.1,21.4) | 3 | 13.02 (4.18,33.61) | 202.8 (39.9,1,030.2) |
| DVT | 3 | 0.01 (0.00,0.04) | 4 | 0.07 (0.02,0.16) | 5.3 (1.0,28.0) | 1 | 0.27 (0.06,1.32) | 21.0 (2.2,201.6) | 0 | NA | NA |
| CPD | 10 | 0.04 (0.02,0.08) | 5 | 0.09 (0.03,0.19) | 2.0 (0.7,5.9) | 1 | 0.21 (0.01,0.89) | 5.0 (0.6,38.9) | 3 | 12.07 (4.23,34.00) | 283.2 (60.6,1,323.0) |
| SLD | 3 | 0.01 (0.00,0.03) | 1 | 0.02 (0.00,0.07) | 1.4 (0.1,13.0) | 1 | 0.20 (0.01,0.87) | 15.7 (1.6,151.1) | 0 | NA | NA |
| Pancreatitis | 6 | 0.02 (0.01,0.05) | 4 | 0.06 (0.02,0.16) | 2.6 (0.7,9.3) | 1 | 0.19 (0.01,0.87) | 7.6 (0.9,63.2) | 0 | NA | NA |
| ESRD | 0 | NA | 0 | NA | NA | 0 | NA | NA | 0 | NA | NA |
| AKD | 3 | 0.01 (0.00,0.04) | 5 | 0.09 (0.04,0.21) | 7.0 (1.5,32.4) | 1 | 0.25 (0.06,1.32) | 19.6 (2.0,188.1) | 0 | NA | NA |
| All-cause mortality | 176 | 0.74 (0.63,0.85) | 87 | 1.56 (1.27,1.93) | 2.1 (1.6,2.8) | 55 | 12.93 (9.78,16.64) | 17.5 (12.9,23.7) | 45 | 172.14 (128.18,229.18) | 232.1 (165.9,324.7) |
| **Age≥65** |  |  |  |  |  |  |  |  |  |  |  |
| Major CVD | 302 | 2.19 (1.95,2.45) | 186 | 7.44 (6.42,8.55) | 3.4 (2.8,4.1) | 81 | 23.18 (18.46,28.58) | 10.5 (8.0,13.8) | 46 | 51.34 (37.81,67.60) | 22.8 (13.9,37.4) |
| Carditis | 4 | 0.02 (0.01,0.06) | 2 | 0.06 (0.01,0.19) | 2.6 (0.5,14.1) | 0 | NA | NA | 0 | NA | NA |
| AF | 61 | 0.39 (0.30,0.49) | 33 | 1.16 (0.82,1.62) | 3.0 (2.0,4.6) | 8 | 2.08 (1.02,3.89) | 5.4 (2.4,11.9) | 3 | 3.03 (1.05,8.48) | 7.9 (3.0,20.8) |
| Flutter | 5 | 0.03 (0.01,0.06) | 0 | 0.00 (0.00,0.13) | 0.0 (0.0,0.0) | 0 | 0.00 (0.00,0.88) | 0.0 (0.0,0.0) | 0 | NA | NA |
| ACD | 94 | 0.58 (0.48,0.71) | 50 | 1.69 (1.26,2.20) | 2.9 (2.0,4.1) | 31 | 7.36 (5.05,10.26) | 12.5 (8.0,19.4) | 15 | 14.49 (8.59,23.24) | 24.1 (14.1,41.3) |
| DVT | 11 | 0.07 (0.04,0.12) | 8 | 0.27 (0.14,0.53) | 4.0 (1.6,10.1) | 4 | 1.02 (0.38,2.42) | 14.8 (5.6,39.3) | 1 | 1.26 (0.22,5.13) | 18.2 (5.0,65.9) |
| CPD | 18 | 0.11 (0.07,0.18) | 16 | 0.55 (0.32,0.85) | 4.8 (2.4,9.8) | 2 | 0.56 (0.15,1.77) | 4.9 (1.6,15.1) | 2 | 2.32 (0.60,6.96) | 20.1 (3.6,112.0) |
| SLD | 5 | 0.03 (0.01,0.07) | 9 | 0.29 (0.14,0.53) | 9.2 (3.0,28.4) | 0 | NA | NA | 0 | NA | NA |
| Pancreatitis | 14 | 0.09 (0.05,0.14) | 7 | 0.24 (0.12,0.48) | 2.8 (1.1,7.3) | 2 | 0.52 (0.15,1.71) | 6.0 (1.0,38.0) | 1 | 0.57 (0.02,3.40) | 6.5 (1.4,29.4) |
| ESRD | 9 | 0.05 (0.03,0.10) | 4 | 0.12 (0.04,0.29) | 2.2 (0.6,8.4) | 2 | 0.44 (0.06,1.31) | 8.2 (2.8,24.2) | 1 | 0.85 (0.02,3.39) | 15.9 (3.3,75.5) |
| AKD | 27 | 0.17 (0.11,0.24) | 23 | 0.79 (0.52,1.16) | 4.7 (2.6,8.3) | 5 | 1.29 (0.52,2.78) | 7.6 (3.5,16.4) | 7 | 6.16 (2.64,12.27) | 36.2 (13.7,95.5) |
| All-cause mortality | 1,541 | 9.38 (8.92,9.85) | 1,442 | 48.04 (45.60,50.56) | 5.1 (4.7,5.5) | 681 | 159.96 (148.29,172.32) | 17.0 (15.7,18.4) | 592 | 541.26 (499.25,586.48) | 56.7 (50.1,64.3) |

Note: :Major CVD: composite outcomes of heart failure, stroke and coronary artery disease; AF: Atrial fibrillation; ACD: Acute coronary disorder; DVT: Deep vein thrombosis; CPD: Chronic pulmonary disease; SLD: Severe liver disease; ESRD: End stage renal disease; AKD: Acute kidney disease; NA: Not available due to insufficient number. Hazard Ratio was obtained by Cox regression adjusted with weighting.

**Supplementary Table 6. Hazard ratio of acute COVID-19 composite outcomes compared to the control groups in male subgroup.**

|  | **Control** | | **Mild** | | | **Severe** | | | **Critical** | | |
| --- | --- | --- | --- | --- | --- | --- | --- | --- | --- | --- | --- |
|  | **Events** | **Incidence rate**  **(per 100,000 person per day)** | **Events** | **Incidence rate**  **(per 100,000 person per day)** | **HR** | **Events** | **Incidence rate**  **(per 100,000 person per day)** | **HR** | **Events** | **Incidence rate**  **(per 100,000 person per day)** | **HR** |
| **Age ≤40** |  |  |  |  |  |  |  |  |  |  |  |
| Major CVD | 11 | 0.07 (0.04,0.12) | 4 | 0.11 (0.04,0.25) | 1.5 (0.5,4.3) | 1 | 0.27 (0.01,1.44) | 3.8 (0.5,29.7) | 3 | 20.48 (6.50,52.26) | 288.1 (78.8,1,053.5) |
| Carditis | 5 | 0.03 (0.01,0.06) | 1 | 0.02 (0.00,0.09) | 0.7 (0.1,6.3) | 0 | NA | NA | 0 | NA | NA |
| AF | 1 | 0.01 (0.00,0.02) | 0 | NA | NA | 0 | NA | NA | 0 | NA | NA |
| Flutter | 0 | NA | 0 | NA | NA | 0 | NA | NA | 0 | NA | NA |
| ACD | 2 | 0.01 (0.00,0.04) | 3 | 0.06 (0.02,0.18) | 4.8 (0.8,29.0) | 0 | NA | NA | 0 | NA | NA |
| DVT | 0 | NA | 1 | 0.02 (0.00,0.09) | NA | 0 | NA | NA | 0 | NA | NA |
| CPD | 7 | 0.04 (0.02,0.08) | 6 | 0.16 (0.07,0.33) | 3.6 (1.2,10.9) | 0 | NA | NA | 0 | NA | NA |
| SLD | 0 | NA | 0 | NA | NA | 0 | NA | NA | 0 | NA | NA |
| Pancreatitis | 4 | 0.03 (0.01,0.06) | 1 | 0.02 (0.00,0.09) | 0.8 (0.1,7.3) | 0 | NA | NA | 2 | 14.40 (3.69,43.13) | NA |
| ESRD | 0 | NA | 0 | NA | NA | 0 | NA | NA | 0 | NA | NA |
| AKD | 0 | NA | 0 | NA | NA | 0 | NA | NA | 1 | 5.55 (0.15,21.77) | NA |
| All-cause mortality | 27 | 0.16 (0.11,0.24) | 6 | 0.15 (0.07,0.32) | 0.9 (0.3,2.4) | 13 | 5.01 (2.69,8.14) | 30.5 (16.0,58.4) | 12 | 68.58 (36.47,115.76) | 417.1 (198.2,877.5) |
| **Age (40-65)** |  |  |  |  |  |  |  |  |  |  |  |
| Major CVD | 221 | 1.38 (1.21,1.57) | 103 | 2.75 (2.27,3.33) | 2.0 (1.6,2.6) | 25 | 9.47 (6.15,13.58) | 6.9 (4.4,10.7) | 35 | 99.54 (71.38,137.80) | 71.6 (43.9,116.8) |
| Carditis | 3 | 0.02 (0.01,0.05) | 1 | 0.02 (0.00,0.09) | 1.2 (0.1,11.2) | 0 | NA | NA | 0 | NA | NA |
| AF | 16 | 0.10 (0.06,0.15) | 6 | 0.14 (0.06,0.29) | 1.5 (0.5,4.1) | 1 | 0.30 (0.01,1.31) | 3.1 (0.4,23.6) | 0 | NA | NA |
| Flutter | 4 | 0.02 (0.01,0.06) | 2 | 0.05 (0.01,0.14) | 1.9 (0.3,10.5) | 0 | NA | NA | 0 | NA | NA |
| ACD | 113 | 0.67 (0.55,0.79) | 41 | 1.02 (0.74,1.37) | 1.5 (1.1,2.2) | 9 | 3.40 (1.72,6.12) | 5.1 (2.5,10.5) | 14 | 36.91 (20.21,58.70) | 55.2 (24.5,124.5) |
| DVT | 2 | 0.01 (0.00,0.03) | 2 | 0.04 (0.01,0.14) | 3.8 (0.5,27.0) | 0 | NA | NA | 0 | NA | NA |
| CPD | 14 | 0.08 (0.04,0.13) | 11 | 0.29 (0.16,0.50) | 3.5 (1.6,7.7) | 1 | 0.53 (0.09,2.00) | 6.6 (1.5,29.6) | 0 | NA | NA |
| SLD | 7 | 0.04 (0.02,0.08) | 6 | 0.14 (0.05,0.29) | 3.3 (1.0,10.8) | 2 | 0.55 (0.09,1.97) | 13.1 (2.3,76.0) | 1 | 3.65 (0.62,14.33) | 85.8 (10.6,693.3) |
| Pancreatitis | 9 | 0.05 (0.03,0.10) | 5 | 0.13 (0.05,0.29) | 2.5 (0.7,8.8) | 2 | 0.56 (0.09,1.97) | 10.6 (1.3,83.5) | 2 | 5.80 (1.63,19.04) | 109.6 (14.0,858.7) |
| ESRD | 1 | 0.01 (0.00,0.02) | 1 | 0.02 (0.00,0.09) | 4.1 (0.3,66.3) | 0 | NA | NA | 0 | NA | NA |
| AKD | 6 | 0.03 (0.01,0.07) | 6 | 0.14 (0.05,0.29) | 4.2 (1.4,13.2) | 1 | 0.21 (0.01,1.31) | 6.3 (0.8,52.0) | 1 | 1.87 (0.07,9.57) | 53.7 (6.5,445.6) |
| All-cause mortality | 304 | 1.76 (1.57,1.96) | 158 | 3.92 (3.33,4.55) | 2.2 (1.8,2.8) | 68 | 24.02 (18.89,30.33) | 13.6 (10.8,17.3) | 73 | 186.64 (146.71,232.48) | 105.6 (80.3,138.8) |
| **Age≥65** |  |  |  |  |  |  |  |  |  |  |  |
| Major CVD | 331 | 3.03 (2.72,3.37) | 203 | 9.34 (8.12,10.69) | 3.1 (2.6,3.7) | 64 | 16.87 (13.03,21.32) | 5.5 (4.1,7.4) | 115 | 93.68 (77.84,112.10) | 29.8 (19.8,44.7) |
| Carditis | 4 | 0.03 (0.01,0.07) | 1 | 0.03 (0.00,0.13) | 1.0 (0.1,9.3) | 1 | 0.17 (0.01,0.76) | 5.7 (0.6,51.0) | 0 | NA | NA |
| AF | 47 | 0.36 (0.27,0.47) | 34 | 1.31 (0.93,1.81) | 3.6 (2.3,5.7) | 8 | 1.82 (0.89,3.42) | 5.0 (2.5,10.0) | 2 | 1.40 (0.41,4.73) | 3.8 (1.4,10.3) |
| Flutter | 7 | 0.05 (0.03,0.11) | 6 | 0.21 (0.08,0.43) | 4.1 (1.3,13.1) | 0 | NA | NA | 0 | NA | 0.0 (0.0,0.0) |
| ACD | 154 | 1.15 (0.97,1.34) | 72 | 2.68 (2.11,3.36) | 2.3 (1.7,3.1) | 24 | 5.12 (3.28,7.37) | 4.4 (3.0,6.6) | 56 | 36.04 (27.55,46.41) | 30.4 (18.7,49.4) |
| DVT | 5 | 0.04 (0.02,0.08) | 8 | 0.31 (0.15,0.57) | 8.4 (2.7,26.0) | 1 | 0.25 (0.05,1.15) | 6.7 (1.2,38.3) | 4 | 2.47 (1.00,6.33) | 66.6 (13.7,325.1) |
| CPD | 4 | 0.03 (0.01,0.07) | 2 | 0.06 (0.01,0.20) | 2.1 (0.2,19.1) | 2 | 0.50 (0.13,1.49) | 16.5 (3.3,81.7) | 2 | 0.96 (0.15,3.41) | 31.1 (3.4,284.4) |
| SLD | 13 | 0.09 (0.05,0.15) | 6 | 0.22 (0.08,0.42) | 2.3 (0.9,6.2) | 0 | NA | NA | 6 | 3.70 (1.37,7.26) | 39.2 (11.5,133.7) |
| Pancreatitis | 5 | 0.04 (0.02,0.08) | 5 | 0.19 (0.08,0.42) | 4.7 (1.1,19.8) | 3 | 0.58 (0.13,1.49) | 14.7 (3.2,67.5) | 2 | 1.37 (0.38,4.44) | 34.0 (10.2,113.5) |
| ESRD | 39 | 0.28 (0.20,0.38) | 24 | 0.89 (0.59,1.31) | 3.1 (1.8,5.4) | 13 | 2.70 (1.45,4.38) | 9.4 (5.0,17.8) | 21 | 13.31 (8.72,20.30) | 45.2 (17.1,119.4) |
| AKD | 1,760 | 12.68 (12.09,13.28) | 1,665 | 60.17 (57.33,63.11) | 4.7 (4.4,5.1) | 952 | 195.18 (183.02,207.83) | 15.3 (14.2,16.5) | 1,065 | 651.56 (612.46,691.27) | 50.4 (46.0,55.2) |
| All-cause mortality | 617 | 5.97 (5.51,6.45) | 425 | 19.93 (18.11,21.90) | 3.3 (2.9,3.8) | 279 | 105.83 (94.13,119.00) | 17.7 (15.5,20.1) | 290 | 477.56 (425.23,535.19) | 78.9 (66.4,93.8) |

Note: AF: : Major CVD: composite outcomes of heart failure, stroke and coronary artery disease; Atrial fibrillation; ACD: Acute coronary disorder; DVT: Deep vein thrombosis; CPD: Chronic pulmonary disease; SLD: Severe liver disease; ESRD: End stage renal disease; AKD: Acute kidney disease; NA: Not available due to insufficient number. Hazard Ratio was obtained by Cox regression adjusted with weighting.

**Supplementary Table 7. Hazard ratio of acute COVID-19 composite outcomes compared to the control groups in fully vaccination subgroup.**

|  | **Control** | | **Mild** | | | **Severe** | | | **Critical** | | |
| --- | --- | --- | --- | --- | --- | --- | --- | --- | --- | --- | --- |
|  | **Events** | **Incidence rate**  **(per 100,000 person per day)** | **Events** | **Incidence rate**  **(per 100,000 person per day)** | **HR** | **Events** | **Incidence rate**  **(per 100,000 person per day)** | **HR** | **Events** | **Incidence rate**  **(per 100,000 person per day)** | **HR** |
| **Age ≤40** |  |  |  |  |  |  |  |  |  |  |  |
| Major CVD | 5 | 0.03 (0.01,0.08) | 4 | 0.09 (0.03,0.22) | 2.6 (0.7,9.0) | 0 | NA | NA | 3 | 23.02 (8.30,66.76) | 656.6 (135.6,3,178.8) |
| Carditis | 4 | 0.03 (0.01,0.06) | 1 | 0.03 (0.01,0.14) | 1.3 (0.1,11.2) | 0 | NA | NA | 0 | NA | NA |
| AF | 0 | NA | 1 | 0.02 (0.00,0.09) | NA | 0 | NA | NA | 0 | NA | NA |
| Flutter | 0 | NA | 1 | 0.02 (0.00,0.09) | NA | 0 | NA | NA | 0 | NA | NA |
| ACD | 2 | 0.01 (0.00,0.05) | 2 | 0.05 (0.01,0.14) | 3.2 (0.5,23.0) | 0 | NA | NA | 0 | NA | NA |
| DVT | 1 | 0.01 (0.00,0.04) | 1 | 0.02 (0.00,0.09) | 3.4 (0.2,53.6) | 0 | NA | NA | 0 | NA | NA |
| CPD | 6 | 0.04 (0.02,0.08) | 2 | 0.05 (0.01,0.14) | 1.2 (0.2,6.0) | 0 | NA | NA | 0 | NA | NA |
| SLD | 0 | NA | 0 | NA | NA | 0 | NA | NA | 1 | 6.31 (0.19,27.94) | NA |
| Pancreatitis | 4 | 0.03 (0.01,0.07) | 4 | 0.09 (0.03,0.22) | 3.3 (0.8,13.1) | 0 | NA | NA | 0 | NA | NA |
| ESRD | 0 | NA | 0 | NA | NA | 0 | NA | NA | 3 | 22.72 (4.70,54.93) | NA |
| AKD | 1 | 0.01 (0.00,0.04) | 0 | NA | NA | 0 | NA | NA | 0 | NA | NA |
| All-cause mortality | 12 | 0.08 (0.05,0.14) | 2 | 0.04 (0.01,0.14) | 0.5 (0.1,2.4) | 5 | 1.87 (0.78,4.12) | 22.3 (6.3,79.0) | 5 | 36.06 (12.29,77.54) | 428.8 (125.8,1,461.1) |
| **Age (40-65)** |  |  |  |  |  |  |  |  |  |  |  |
| Major CVD | 105 | 0.70 (0.57,0.84) | 52 | 1.32 (0.99,1.71) | 1.9 (1.3,2.6) | 15 | 4.68 (2.76,7.47) | 6.7 (3.9,11.5) | 16 | 73.56 (44.46,116.64) | 104.0 (55.9,193.4) |
| Carditis | 3 | 0.02 (0.01,0.06) | 1 | 0.03 (0.01,0.14) | 1.3 (0.1,12.7) | 0 | NA | NA | 0 | NA | NA |
| AF | 16 | 0.10 (0.06,0.17) | 3 | 0.07 (0.02,0.18) | 0.7 (0.2,2.4) | 1 | 0.33 (0.07,1.62) | 3.2 (0.4,24.4) | 0 | NA | NA |
| Flutter | 2 | 0.01 (0.00,0.05) | 2 | 0.05 (0.01,0.14) | 3.7 (0.5,26.1) | 0 | NA | NA | 0 | NA | NA |
| ACD | 38 | 0.25 (0.17,0.33) | 15 | 0.38 (0.23,0.61) | 1.6 (0.9,2.8) | 4 | 1.17 (0.47,2.98) | 4.8 (1.7,13.5) | 5 | 20.46 (6.94,43.76) | 83.0 (31.1,221.3) |
| DVT | 3 | 0.02 (0.00,0.05) | 2 | 0.05 (0.01,0.14) | 2.5 (0.4,15.0) | 0 | NA | NA | 0 | NA | NA |
| CPD | 6 | 0.04 (0.01,0.08) | 9 | 0.23 (0.12,0.42) | 5.9 (2.1,16.5) | 2 | 0.55 (0.07,1.64) | 14.1 (2.8,70.6) | 0 | NA | NA |
| SLD | 5 | 0.03 (0.01,0.07) | 3 | 0.08 (0.03,0.21) | 2.4 (0.6,9.9) | 2 | 0.44 (0.07,1.61) | 13.5 (2.5,72.2) | 1 | 3.44 (0.11,15.59) | 105.0 (12.3,893.8) |
| Pancreatitis | 2 | 0.01 (0.00,0.05) | 4 | 0.09 (0.03,0.21) | 7.2 (1.3,39.6) | 1 | 0.29 (0.01,1.07) | 22.2 (2.0,244.8) | 0 | NA | NA |
| ESRD | 0 | NA | 0 | NA | NA | 0 | NA | NA | 0 | NA | NA |
| AKD | 2 | 0.01 (0.00,0.05) | 2 | 0.05 (0.01,0.14) | 3.5 (0.5,24.6) | 0 | NA | NA | 0 | NA | NA |
| All-cause mortality | 132 | 0.85 (0.71,1.00) | 46 | 1.12 (0.82,1.47) | 1.3 (0.9,1.9) | 29 | 8.23 (5.60,11.67) | 9.7 (6.7,14.0) | 55 | 231.15 (174.58,296.88) | 271.2 (188.9,389.4) |
| **Age≥65** |  |  |  |  |  |  |  |  |  |  |  |
| Major CVD | 189 | 2.20 (1.90,2.53) | 118 | 6.69 (5.57,7.99) | 3.0 (2.4,3.9) | 51 | 23.75 (18.07,31.20) | 10.7 (7.7,15.0) | 38 | 77.74 (55.35,104.97) | 34.4 (21.3,55.5) |
| Carditis | 1 | 0.01 (0.00,0.05) | 3 | 0.16 (0.05,0.41) | 16.3 (1.7,157.4) | 1 | 0.25 (0.01,1.40) | 25.1 (1.6,403.3) | 0 | NA | NA |
| AF | 39 | 0.39 (0.29,0.54) | 19 | 0.94 (0.60,1.45) | 2.4 (1.4,4.2) | 3 | 1.08 (0.25,2.87) | 2.8 (0.8,9.4) | 2 | 3.46 (0.42,9.70) | 8.8 (2.6,30.0) |
| Flutter | 3 | 0.03 (0.01,0.09) | 0 | 0.00 (0.00,0.18) | NA | 0 | NA | 0.0 (0.0,0.0) | 0 | NA | NA |
| ACD | 77 | 0.76 (0.61,0.95) | 31 | 1.48 (1.01,2.05) | 1.9 (1.3,3.0) | 17 | 6.44 (3.86,10.14) | 8.4 (4.7,14.9) | 15 | 26.36 (15.56,42.11) | 33.8 (17.1,66.8) |
| DVT | 7 | 0.07 (0.03,0.13) | 4 | 0.17 (0.05,0.41) | 2.5 (0.6,9.6) | 2 | 0.69 (0.09,2.12) | 10.3 (2.5,42.4) | 2 | 3.80 (1.03,11.98) | 55.3 (11.7,261.8) |
| CPD | 22 | 0.23 (0.15,0.33) | 10 | 0.51 (0.27,0.90) | 2.3 (1.1,4.8) | 2 | 0.68 (0.10,2.23) | 3.0 (0.9,10.1) | 2 | 4.09 (1.08,12.61) | 18.1 (3.7,87.4) |
| SLD | 3 | 0.03 (0.01,0.08) | 0 | NA | NA | 1 | 0.26 (0.01,1.40) | 8.4 (0.9,80.8) | 0 | NA | NA |
| Pancreatitis | 8 | 0.08 (0.03,0.14) | 3 | 0.15 (0.05,0.41) | 2.0 (0.5,7.5) | 0 | NA | NA | 0 | NA | NA |
| ESRD | 5 | 0.05 (0.02,0.11) | 1 | 0.06 (0.01,0.26) | 1.2 (0.1,9.9) | 1 | 0.48 (0.09,2.12) | 9.3 (1.5,55.8) | 0 | NA | NA |
| AKD | 20 | 0.19 (0.12,0.29) | 9 | 0.41 (0.19,0.74) | 2.1 (0.9,4.9) | 3 | 1.28 (0.42,3.36) | 6.7 (2.5,17.9) | 7 | 11.48 (4.77,22.14) | 58.1 (17.6,191.9) |
| All-cause mortality | 617 | 5.97 (5.51,6.45) | 425 | 19.93 (18.11,21.90) | 3.3 (2.9,3.8) | 279 | 105.83 (94.13,119.00) | 17.7 (15.5,20.1) | 290 | 477.56 (425.23,535.19) | 78.9 (66.4,93.8) |

Note: AF: : Major CVD: composite outcomes of heart failure, stroke and coronary artery disease; Atrial fibrillation; ACD: Acute coronary disorder; DVT: Deep vein thrombosis; CPD: Chronic pulmonary disease; SLD: Severe liver disease; ESRD: End stage renal disease; AKD: Acute kidney disease; NA: Not available due to insufficient number. Hazard Ratio was obtained by Cox regression adjusted with weighting.

**Supplementary Table 8. Hazard ratio of acute COVID-19 composite outcomes compared to the control groups in non-fully vaccination subgroup.**

|  | **Control** | | **Mild** | | | **Severe** | | | **Critical** | | |
| --- | --- | --- | --- | --- | --- | --- | --- | --- | --- | --- | --- |
|  | **Events** | **Incidence rate**  **(per 100,000 person per day)** | **Events** | **Incidence rate**  **(per 100,000 person per day)** | **HR** | **Events** | **Incidence rate**  **(per 100,000 person per day)** | **HR** | **Events** | **Incidence rate**  **(per 100,000 person per day)** | **HR** |
| **Age ≤40** |  |  |  |  |  |  |  |  |  |  |  |
| Major CVD | 7 | 0.05 (0.02,0.10) | 4 | 0.03 (0.00,0.16) | 2.5 (0.6,9.6) | 2 | 0.83 (0.15,3.35) | 10.3 (2.5,42.4) | 2 | 16.96 (4.08,47.68) | 55.3 (11.7,261.8) |
| Carditis | 2 | 0.02 (0.01,0.06) | 1 | 0.04 (0.00,0.16) | 2.1 (0.2,23.4) | 0 | NA | NA | 0 | NA | NA |
| AF | 1 | 0.01 (0.00,0.03) | 0 | NA | NA | 0 | NA | NA | 1 | NA | 137.0 (9.0,2,080.4) |
| Flutter | 0 | NA | 0 | NA | NA | 0 | NA | NA | 0 | NA | NA |
| ACD | 0 | 0.01 (0.00,0.05) | 0 | 0.03 (0.00,0.16) | NA | 0 | NA | NA | 0 | NA | NA |
| DVT | 22 | NA | 10 | NA | 2.3 (1.1,4.8) | 2 | NA | 3.0 (0.9,10.1) | 2 | NA | 18.1 (3.7,87.4) |
| CPD | 7 | 0.02 (0.01,0.07) | 4 | 0.23 (0.10,0.50) | 2.5 (0.6,9.6) | 2 | NA | 10.3 (2.5,42.4) | 2 | NA | 55.3 (11.7,261.8) |
| SLD | 2 | NA | 1 | NA | 2.1 (0.2,23.4) | 0 | NA | NA | 0 | NA | NA |
| Pancreatitis | 1 | NA | 0 | NA | NA | 0 | NA | NA | 1 | NA | 137.0 (9.0,2,080.4) |
| ESRD | 0 | 0.01 (0.00,0.03) | 0 | 0.00 (0.00,0.16) | NA | 0 | NA | NA | 0 | NA | NA |
| AKD | 0 | NA | 0 | NA | NA | 0 | NA | NA | 0 | 18.33 (4.05,47.28) | NA |
| All-cause mortality | 22 | 0.25 (0.17,0.36) | 10 | 0.25 (0.09,0.50) | 2.3 (1.1,4.8) | 2 | 8.67 (5.02,14.06) | 3.0 (0.9,10.1) | 2 | 78.28 (44.77,135.58) | 18.1 (3.7,87.4) |
| **Age (40-65)** | 3 |  | 0 |  | NA | 1 |  | 8.4 (0.9,80.8) | 0 |  | NA |
| Major CVD | 8 | 1.11 (0.88,1.38) | 3 | 3.03 (2.18,4.17) | 2.0 (0.5,7.5) | 0 | 5.96 (2.62,12.17) | NA | 0 | 37.00 (16.30,68.07) | NA |
| Carditis | 5 | 0.04 (0.01,0.12) | 1 | NA | 1.2 (0.1,9.9) | 1 | NA | 9.3 (1.5,55.8) | 0 | NA | NA |
| AF | 20 | 0.03 (0.01,0.10) | 9 | 0.18 (0.05,0.57) | 2.1 (0.9,4.9) | 3 | NA | 6.7 (2.5,17.9) | 7 | NA | 58.1 (17.6,191.9) |
| Flutter | 7 | 0.01 (0.00,0.05) | 4 | 0.00 (0.00,0.29) | 2.5 (0.6,9.6) | 2 | NA | 10.3 (2.5,42.4) | 2 | NA | 55.3 (11.7,261.8) |
| ACD | 2 | 0.49 (0.35,0.67) | 1 | 0.83 (0.43,1.46) | 2.1 (0.2,23.4) | 0 | 1.86 (0.54,6.32) | NA | 0 | 13.57 (4.72,37.97) | NA |
| DVT | 1 | 0.01 (0.00,0.05) | 0 | 0.18 (0.05,0.57) | NA | 0 | 1.13 (0.21,4.82) | NA | 1 | NA | 137.0 (9.0,2,080.4) |
| CPD | 8 | 0.05 (0.02,0.12) | 3 | 0.12 (0.02,0.44) | 2.0 (0.5,7.5) | 0 | NA | NA | 0 | 17.18 (4.82,38.75) | NA |
| SLD | 5 | 0.04 (0.01,0.12) | 1 | 0.06 (0.00,0.29) | 1.2 (0.1,9.9) | 1 | NA | 9.3 (1.5,55.8) | 0 | 0.00 (0.00,15.72) | NA |
| Pancreatitis | 20 | 0.04 (0.01,0.10) | 9 | 0.23 (0.05,0.57) | 2.1 (0.9,4.9) | 3 | NA | 6.7 (2.5,17.9) | 7 | 0.00 (0.00,15.95) | 58.1 (17.6,191.9) |
| ESRD | 7 | 0.01 (0.00,0.08) | 4 | NA | 2.5 (0.6,9.6) | 2 | NA | 10.3 (2.5,42.4) | 2 | 0.56 (0.11,15.76) | 55.3 (11.7,261.8) |
| AKD | 2 | 0.01 (0.00,0.08) | 1 | 0.26 (0.09,0.69) | 2.1 (0.2,23.4) | 0 | NA | NA | 0 | 3.82 (0.11,16.01) | NA |
| All-cause mortality | 1 | 3.93 (3.49,4.40) | 0 | 9.86 (8.27,11.71) | NA | 0 | 67.92 (53.85,83.81) | NA | 1 | 460.78 (380.86,554.88) | 137.0 (9.0,2,080.4) |
| **Age≥65** | 0 |  | 0 |  | NA | 0 |  | NA | 0 |  | NA |
| Major CVD | 0 | 3.37 (2.97,3.80) | 0 | 13.65 (11.79,15.72) | NA | 0 | 26.68 (21.56,32.56) | NA | 0 | 64.30 (51.19,79.91) | NA |
| Carditis | 22 | 0.05 (0.02,0.12) | 10 | NA | 2.3 (1.1,4.8) | 2 | NA | 3.0 (0.9,10.1) | 2 | NA | 18.1 (3.7,87.4) |
| AF | 3 | 0.41 (0.29,0.55) | 0 | 1.40 (0.92,2.05) | NA | 1 | 3.33 (1.95,5.46) | 8.4 (0.9,80.8) | 0 | 2.62 (1.06,6.66) | NA |
| Flutter | 8 | 0.02 (0.01,0.07) | 3 | 0.10 (0.01,0.32) | 2.0 (0.5,7.5) | 0 | NA | NA | 0 | NA | NA |
| ACD | 5 | 1.12 (0.92,1.35) | 1 | 2.93 (2.23,3.85) | 1.2 (0.1,9.9) | 1 | 10.42 (7.72,13.71) | 9.3 (1.5,55.8) | 0 | 30.49 (22.96,40.07) | NA |
| DVT | 20 | 0.07 (0.03,0.15) | 9 | 0.69 (0.38,1.16) | 2.1 (0.9,4.9) | 3 | 1.01 (0.35,2.21) | 6.7 (2.5,17.9) | 7 | 2.26 (0.65,5.25) | 58.1 (17.6,191.9) |
| CPD | 0 | 0.14 (0.07,0.22) | 0 | 1.39 (0.91,2.04) | NA | 0 | 1.11 (0.38,2.37) | NA | 0 | 2.01 (0.70,5.65) | NA |
| SLD | 22 | 0.05 (0.02,0.12) | 10 | 0.49 (0.23,0.87) | 2.3 (1.1,4.8) | 2 | 0.39 (0.05,1.20) | 3.0 (0.9,10.1) | 2 | 0.33 (0.02,2.19) | 18.1 (3.7,87.4) |
| Pancreatitis | 3 | 0.09 (0.04,0.16) | 0 | 0.45 (0.23,0.88) | NA | 1 | NA | 8.4 (0.9,80.8) | 0 | 5.39 (2.47,9.45) | NA |
| ESRD | 8 | 0.08 (0.03,0.15) | 3 | 0.27 (0.09,0.57) | 2.0 (0.5,7.5) | 0 | 0.67 (0.23,1.89) | NA | 0 | 2.89 (0.97,6.12) | NA |
| AKD | 5 | 0.34 (0.24,0.47) | 1 | 1.51 (1.00,2.15) | 1.2 (0.1,9.9) | 1 | 4.25 (2.70,6.55) | 9.3 (1.5,55.8) | 0 | 7.88 (4.30,13.03) | NA |
| All-cause mortality | 20 | 24.57 (23.61,25.56) | 9 | 124.25 (119.19,129.46) | 2.1 (0.9,4.9) | 3 | 412.71 (394.54,431.41) | 6.7 (2.5,17.9) | 7 | 1,280.98 (1,227.99,1,336.04) | 58.1 (17.6,191.9) |

Note: : Major CVD: composite outcomes of heart failure, stroke and coronary artery disease; AF: Atrial fibrillation; ACD: Acute coronary disorder; DVT: Deep vein thrombosis; CPD: Chronic pulmonary disease; SLD: Severe liver disease; ESRD: End stage renal disease; AKD: Acute kidney disease; NA: Not available due to insufficient number. Hazard Ratio was obtained by Cox regression adjusted with weighting.

**Supplementary Table 9. Hazard ratio of post-acute COVID-19 composite outcomes compared to the control groups in female subgroup.**

|  | **Control** | | **Mild** | | | **Severe** | | | **Critical** | | |
| --- | --- | --- | --- | --- | --- | --- | --- | --- | --- | --- | --- |
|  | **Events** | **Incidence rate**  **(per 100,000 person per year)** | **Events** | **Incidence rate**  **(per 100,000 person per year)** | **HR** | **Events** | **Incidence rate**  **(per 100,000 person per year)** | **HR** | **Events** | **Incidence rate**  **(per 100,000 person per year)** | **HR** |
| **Age ≤40** |  |  |  |  |  |  |  |  |  |  |  |
| Major CVD | 72 | 1.8 (1.5,2.3) | 57 | 1.5 (1.1,1.9) | 0.8 (0.4,1.5) | 163 | 3.9 (3.3,4.5) | 2.2 (0.5,9.3) | 0 | NA | NA |
| Carditis | 24 | 0.6 (0.4,0.9) | 15 | 0.4 (0.2,0.6) | 0.6 (0.2,2.1) | 128 | 3.0 (2.5,3.6) | 5.2 (1.1,25.4) | 0 | NA | NA |
| AF | 8 | 0.2 (0.1,0.4) | 9 | 0.2 (0.1,0.4) | 1.2 (0.2,5.9) | 0 | NA | NA | 0 | NA | NA |
| Flutter | 5 | 0.1 (0.1,0.3) | 0 | NA | NA | 0 | NA | NA | 0 | NA | NA |
| ACD | 4 | 0.1 (0.0,0.2) | 4 | 0.1 (0.0,0.3) | 1.1 (0.1,10.3) | 0 | NA | NA | 0 | NA | NA |
| DVT | 17 | 0.4 (0.3,0.7) | 14 | 0.4 (0.2,0.6) | 0.8 (0.2,2.8) | 0 | NA | NA | 0 | NA | NA |
| CPD | 112 | 2.9 (2.4,3.5) | 117 | 3.0 (2.5,3.6) | 1.0 (0.6,1.7) | 290 | 7.0 (6.2,7.8) | 2.4 (0.8,7.7) | 804 | 21.0 (19.6,22.5) | 7.3 (1.0,52.9) |
| SLD | 3 | 0.1 (0.0,0.2) | 13 | 0.3 (0.2,0.5) | 4.6 (0.6,33.6) | 64 | 1.5 (1.2,1.9) | 22.7 (2.0,261.3) | 0 | NA | NA |
| Pancreatitis | 26 | 0.7 (0.4,0.9) | 21 | 0.5 (0.3,0.8) | 0.8 (0.3,2.4) | 0 | NA | NA | 0 | NA | NA |
| ESRD | 8 | 0.2 (0.1,0.4) | 6 | 0.1 (0.1,0.3) | 0.7 (0.1,6.0) | 0 | NA | NA | 0 | NA | NA |
| AKD | 6 | 0.2 (0.1,0.3) | 18 | 0.5 (0.3,0.7) | 2.9 (0.7,12.3) | 54 | 1.3 (1.0,1.7) | 8.2 (1.0,68.5) | 0 | NA | NA |
| All-cause mortality | 134 | 3.4 (2.9,4.0) | 109 | 2.8 (2.3,3.4) | 0.8 (0.5,1.3) | 203 | 4.8 (4.2,5.6) | 1.5 (0.6,3.6) | 1,832 | 46.9 (44.8,49.1) | 13.5 (3.3,55.1) |
| **Age (40-65)** |  |  |  |  |  |  |  |  |  |  |  |
| Major CVD | 1,113 | 22.1 (20.9,23.5) | 1,214 | 24.5 (23.1,25.9) | 1.1 (1.0,1.3) | 1,642 | 30.4 (29.0,31.9) | 1.4 (0.9,2.1) | 1,299 | 28.3 (26.8,29.9) | 1.3 (0.4,4.0) |
| Carditis | 19 | 0.4 (0.2,0.6) | 27 | 0.5 (0.4,0.8) | 1.5 (0.5,4.2) | 0 | NA | NA | 0 | NA | NA |
| AF | 113 | 2.2 (1.8,2.6) | 139 | 2.7 (2.3,3.2) | 1.2 (0.8,2.0) | 46 | 0.8 (0.6,1.1) | 0.4 (0.1,2.8) | 0 | NA | NA |
| Flutter | 13 | 0.2 (0.1,0.4) | 29 | 0.6 (0.4,0.8) | 2.3 (0.7,7.6) | 0 | NA | NA | 0 | NA | NA |
| ACD | 189 | 3.7 (3.2,4.2) | 241 | 4.7 (4.2,5.4) | 1.3 (0.9,1.8) | 422 | 7.6 (6.9,8.4) | 2.0 (0.8,4.6) | 31 | 0.7 (0.5,0.9) | 0.2 (0.0,1.3) |
| DVT | 76 | 1.5 (1.2,1.8) | 46 | 0.9 (0.7,1.2) | 0.6 (0.3,1.3) | 81 | 1.5 (1.2,1.8) | 1.0 (0.1,7.6) | 0 | NA | NA |
| CPD | 128 | 2.5 (2.1,3.0) | 189 | 3.7 (3.2,4.3) | 1.5 (1.0,2.2) | 64 | 1.2 (0.9,1.5) | 0.5 (0.1,3.3) | 0 | NA | NA |
| SLD | 26 | 0.5 (0.3,0.7) | 37 | 0.7 (0.5,1.0) | 1.5 (0.6,3.8) | 25 | 0.4 (0.3,0.6) | 0.9 (0.1,6.8) | 0 | NA | NA |
| Pancreatitis | 84 | 1.6 (1.3,2.0) | 99 | 1.9 (1.6,2.3) | 1.2 (0.7,2.1) | 0 | NA | NA | 0 | NA | NA |
| ESRD | 24 | 0.5 (0.3,0.7) | 27 | 0.5 (0.4,0.8) | 1.1 (0.4,3.2) | 51 | 0.9 (0.7,1.2) | 1.9 (0.4,9.6) | 0 | NA | NA |
| AKD | 43 | 0.8 (0.6,1.1) | 39 | 0.8 (0.5,1.0) | 0.9 (0.4,2.1) | 0 | NA | NA | 593 | 12.4 (11.5,13.5) | 14.3 (2.4,84.1) |
| All-cause mortality | 1,301 | 25.2 (23.9,26.6) | 1,526 | 30.0 (28.5,31.5) | 1.2 (1.0,1.4) | 4,452 | 80.4 (78.0,82.8) | 3.2 (2.6,4.1) | 16,856 | 350.1 (344.9,355.4) | 13.9 (8.1,24.0) |
| **Age≥65** |  |  |  |  |  |  |  |  |  |  |  |
| Major CVD | 3,117 | 108.9 (105.1,112.8) | 3,646 | 131.6 (127.4,135.9) | 1.2 (1.1,1.3) | 4,742 | 171.6 (166.8,176.6) | 1.6 (1.3,2.0) | 4,685 | 201.6 (195.9,207.4) | 1.8 (1.2,2.8) |
| Carditis | 40 | 1.2 (0.9,1.6) | 51 | 1.5 (1.2,2.0) | 1.3 (0.6,2.9) | 51 | 1.5 (1.2,2.0) | 1.3 (0.3,5.5) | 0 | NA | NA |
| AF | 705 | 21.5 (20.0,23.2) | 999 | 31.3 (29.4,33.3) | 1.5 (1.2,1.8) | 1,230 | 38.4 (36.3,40.6) | 1.8 (1.2,2.8) | 1,112 | 40.4 (38.1,42.8) | 1.9 (0.7,5.3) |
| Flutter | 42 | 1.2 (0.9,1.7) | 86 | 2.6 (2.1,3.2) | 2.1 (1.1,4.2) | 0 | NA | NA | 0 | NA | NA |
| ACD | 831 | 24.8 (23.1,26.5) | 1,024 | 31.2 (29.4,33.2) | 1.3 (1.0,1.5) | 1,548 | 47.0 (44.7,49.4) | 1.9 (1.3,2.7) | 1,185 | 41.5 (39.2,43.9) | 1.7 (0.9,3.2) |
| DVT | 133 | 3.9 (3.3,4.6) | 218 | 6.5 (5.7,7.4) | 1.7 (1.1,2.5) | 348 | 10.4 (9.4,11.5) | 2.7 (1.4,5.1) | 817 | 28.1 (26.3,30.1) | 7.0 (1.5,31.8) |
| CPD | 216 | 6.5 (5.7,7.5) | 349 | 10.8 (9.8,12.0) | 1.7 (1.2,2.3) | 531 | 16.5 (15.1,18.0) | 2.5 (1.3,5.0) | 263 | 9.5 (8.4,10.7) | 1.5 (0.4,5.4) |
| SLD | 60 | 1.8 (1.4,2.3) | 54 | 1.6 (1.2,2.1) | 0.9 (0.4,1.9) | 36 | 1.1 (0.8,1.5) | 0.6 (0.1,3.0) | 321 | 10.9 (9.8,12.2) | 5.9 (0.8,41.8) |
| Pancreatitis | 153 | 4.5 (3.8,5.2) | 202 | 6.1 (5.3,7.0) | 1.3 (0.9,2.1) | 184 | 5.5 (4.8,6.4) | 1.2 (0.6,2.8) | 191 | 6.6 (5.7,7.5) | 1.4 (0.5,4.6) |
| ESRD | 57 | 1.7 (1.3,2.2) | 65 | 1.9 (1.5,2.5) | 1.2 (0.6,2.4) | 229 | 6.8 (6.0,7.8) | 4.2 (1.6,10.7) | 27 | 0.9 (0.6,1.3) | 0.6 (0.1,4.0) |
| AKD | 329 | 9.7 (8.7,10.8) | 380 | 11.5 (10.4,12.7) | 1.2 (0.9,1.6) | 610 | 18.4 (17.0,19.9) | 1.9 (1.2,3.0) | 1,013 | 35.5 (33.4,37.8) | 3.7 (1.4,9.3) |
| All-cause mortality | 8,218 | 241.0 (235.8,246.2) | 12,486 | 373.9 (367.4,380.5) | 1.5 (1.5,1.6) | 25,088 | 747.3 (738.1,756.6) | 3.1 (2.9,3.4) | 42,590 | 1450.4 (1436.6,1464.2) | 5.9 (5.1,6.9) |

Note: : Major CVD: composite outcomes of heart failure, stroke and coronary artery disease; AF: Atrial fibrillation; ACD: Acute coronary disorder; DVT: Deep vein thrombosis; CPD: Chronic pulmonary disease; SLD: Severe liver disease; ESRD: End stage renal disease; AKD: Acute kidney disease; NA: Not available due to insufficient number. Hazard Ratio was obtained by Cox regression adjusted with weighting.

**Supplementary Table 10. Hazard ratio of post-acute COVID-19 composite outcomes compared to the control groups in male subgroup.**

|  | **Control** | | **Mild** | | | **Severe** | | | **Critical** | | |
| --- | --- | --- | --- | --- | --- | --- | --- | --- | --- | --- | --- |
|  | **Events** | **Incidence rate**  **(per 100,000 person per year)** | **Events** | **Incidence rate**  **(per 100,000 person per year)** | **HR** | **Events** | **Incidence rate**  **(per 100,000 person per year)** | **HR** | **Events** | **Incidence rate**  **(per 100,000 person per year)** | **HR** |
| **Age ≤40** |  |  |  |  |  |  |  |  |  |  |  |
| Major CVD | 156 | 4.5 (3.8,5.2) | 146 | 4.2 (3.5,4.9) | 0.9 (0.6,1.4) | 139 | 3.7 (3.1,4.4) | 0.8 (0.2,2.9) | 0 | NA | NA |
| Carditis | 53 | 1.5 (1.1,2.0) | 59 | 1.7 (1.3,2.2) | 1.1 (0.6,2.1) | 79 | 2.1 (1.7,2.6) | 1.4 (0.2,10.2) | 1,186 | 36.1 (34.1,38.2) | 24.2 (3.3,176.1) |
| AF | 14 | 0.4 (0.2,0.7) | 25 | 0.7 (0.5,1.0) | 1.7 (0.6,5.0) | 73 | 2.0 (1.5,2.4) | 4.7 (0.6,37.4) | 0 | NA | NA |
| Flutter | 1 | 0.0 (0.0,0.2) | 14 | 0.4 (0.2,0.7) | 10.6 (1.1,102.4) | 73 | 1.9 (1.5,2.4) | 50.9 (3.1,832.5) | 0 | NA | NA |
| ACD | 35 | 1.0 (0.7,1.4) | 35 | 1.0 (0.7,1.4) | 1.0 (0.4,2.2) | 0 | NA | NA | 0 | NA | NA |
| DVT | 6 | 0.2 (0.1,0.4) | 5 | 0.1 (0.1,0.3) | 0.8 (0.1,6.7) | 111 | 3.0 (2.5,3.6) | 17.3 (2.0,149.0) | 0 | NA | NA |
| CPD | 173 | 5.0 (4.3,5.8) | 190 | 5.5 (4.8,6.3) | 1.1 (0.7,1.6) | 547 | 14.9 (13.7,16.1) | 3.0 (1.4,6.5) | 0 | NA | NA |
| SLD | 8 | 0.2 (0.1,0.4) | 5 | 0.1 (0.0,0.3) | 0.6 (0.1,4.8) | 0 | NA | NA | 240 | 7.3 (6.4,8.2) | 33.4 (4.0,278.5) |
| Pancreatitis | 27 | 0.8 (0.5,1.1) | 27 | 0.8 (0.5,1.1) | 1.0 (0.4,2.4) | 0 | NA | NA | 0 | NA | NA |
| ESRD | 4 | 0.1 (0.0,0.3) | 0 | NA | NA | 0 | NA | NA | 1,252 | 38.0 (36.0,40.2) | 330.7 (34.4,3,174.5) |
| AKD | 9 | 0.3 (0.1,0.5) | 10 | 0.3 (0.2,0.5) | 1.1 (0.2,5.2) | 0 | NA | NA | 0 | NA | NA |
| All-cause mortality | 203 | 5.8 (5.1,6.7) | 126 | 3.6 (3.0,4.3) | 0.6 (0.4,1.0) | 263 | 7.0 (6.2,7.9) | 1.1 (0.4,2.7) | 3,249 | 98.3 (95.0,101.7) | 17.5 (4.7,64.6) |
| **Age (40-65)** |  |  |  |  |  |  |  |  |  |  |  |
| Major CVD | 2,463 | 71.5 (68.7,74.4) | 2,402 | 70.4 (67.6,73.2) | 1.0 (0.9,1.1) | 2,991 | 82.0 (79.1,84.9) | 1.2 (0.8,1.6) | 3,592 | 116.9 (113.1,120.7) | 1.6 (0.7,3.7) |
| Carditis | 32 | 0.9 (0.6,1.2) | 13 | 0.4 (0.2,0.6) | 0.4 (0.1,1.4) | 92 | 2.3 (1.9,2.8) | 2.8 (0.5,15.7) | 1,178 | 34.7 (32.8,36.7) | 39.3 (9.7,160.1) |
| AF | 224 | 6.1 (5.3,6.9) | 313 | 8.6 (7.7,9.6) | 1.4 (1.0,1.9) | 360 | 9.2 (8.3,10.2) | 1.6 (0.7,3.6) | 182 | 5.4 (4.6,6.2) | 0.9 (0.2,3.2) |
| Flutter | 43 | 1.2 (0.8,1.5) | 44 | 1.2 (0.9,1.6) | 1.0 (0.5,2.2) | 8 | 0.2 (0.1,0.4) | 0.2 (0.0,0.9) | 0 | NA | NA |
| ACD | 1,086 | 29.8 (28.1,31.6) | 1,004 | 27.8 (26.1,29.6) | 0.9 (0.8,1.1) | 1,275 | 32.9 (31.2,34.8) | 1.1 (0.7,1.8) | 1,953 | 58.7 (56.2,61.4) | 1.9 (0.6,5.8) |
| DVT | 63 | 1.7 (1.3,2.2) | 80 | 2.2 (1.7,2.7) | 1.3 (0.7,2.3) | 148 | 3.8 (3.2,4.4) | 2.3 (0.5,9.7) | 0 | NA | NA |
| CPD | 155 | 4.2 (3.6,4.9) | 205 | 5.7 (4.9,6.5) | 1.3 (0.9,2.0) | 245 | 6.3 (5.6,7.2) | 1.6 (0.5,5.0) | 392 | 11.6 (10.5,12.8) | 2.7 (0.4,18.9) |
| SLD | 89 | 2.4 (1.9,2.9) | 82 | 2.2 (1.8,2.8) | 0.9 (0.5,1.7) | 154 | 3.9 (3.3,4.6) | 1.7 (0.6,4.9) | 42 | 1.2 (0.9,1.7) | 0.5 (0.1,3.7) |
| Pancreatitis | 92 | 2.5 (2.0,3.0) | 67 | 1.8 (1.4,2.3) | 0.7 (0.4,1.4) | 111 | 2.8 (2.3,3.4) | 1.2 (0.3,5.4) | 1,164 | 35.0 (33.0,37.0) | 13.4 (1.9,95.2) |
| ESRD | 22 | 0.6 (0.4,0.9) | 18 | 0.5 (0.3,0.7) | 0.8 (0.2,2.7) | 121 | 3.1 (2.6,3.6) | 5.3 (1.2,24.4) | 54 | 1.6 (1.2,2.0) | 2.5 (0.3,18.7) |
| AKD | 101 | 2.7 (2.2,3.3) | 149 | 4.1 (3.4,4.7) | 1.5 (0.9,2.4) | 105 | 2.7 (2.2,3.2) | 1.0 (0.2,4.4) | 0 | NA | NA |
| All-cause mortality | 2,032 | 54.7 (52.4,57.2) | 2,175 | 59.1 (56.7,61.7) | 1.1 (1.0,1.2) | 5,241 | 133.0 (129.4,136.6) | 2.5 (2.0,3.1) | 14,876 | 434.5 (427.6,441.6) | 7.9 (5.5,11.1) |
| **Age≥65** |  |  |  |  |  |  |  |  |  |  |  |
| Major CVD | 3,698 | 158.8 (153.7,164.0) | 4,220 | 186.2 (180.6,191.9) | 1.2 (1.1,1.3) | 5,416 | 247.0 (240.5,253.7) | 1.5 (1.3,1.8) | 3,876 | 213.3 (206.6,220.1) | 1.3 (0.9,2.0) |
| Carditis | 47 | 1.6 (1.2,2.1) | 48 | 1.7 (1.3,2.2) | 1.0 (0.5,2.3) | 49 | 1.7 (1.3,2.2) | 1.1 (0.2,7.8) | 0 | NA | NA |
| AF | 626 | 22.2 (20.5,24.0) | 802 | 29.2 (27.3,31.3) | 1.3 (1.1,1.6) | 1,036 | 38.6 (36.3,41.0) | 1.7 (1.2,2.6) | 263 | 11.4 (10.1,12.9) | 0.5 (0.2,1.3) |
| Flutter | 68 | 2.3 (1.8,2.9) | 91 | 3.2 (2.6,4.0) | 1.4 (0.8,2.4) | 62 | 2.2 (1.7,2.9) | 1.0 (0.3,3.5) | 104 | 4.3 (3.6,5.2) | 1.8 (0.4,7.4) |
| ACD | 1,423 | 49.9 (47.3,52.5) | 1,667 | 59.9 (57.1,62.8) | 1.2 (1.1,1.4) | 1,676 | 61.5 (58.6,64.5) | 1.2 (0.9,1.6) | 1,391 | 58.8 (55.8,62.0) | 1.2 (0.6,2.5) |
| DVT | 82 | 2.8 (2.2,3.5) | 140 | 4.8 (4.1,5.7) | 1.7 (1.0,2.9) | 132 | 4.7 (3.9,5.5) | 1.7 (0.8,3.6) | 638 | 26.1 (24.1,28.2) | 8.9 (2.5,31.4) |
| CPD | 465 | 16.5 (15.1,18.1) | 595 | 21.9 (20.2,23.7) | 1.3 (1.0,1.7) | 953 | 36.1 (33.9,38.4) | 2.2 (1.5,3.2) | 779 | 34.5 (32.1,37.0) | 2.0 (0.8,5.0) |
| SLD | 80 | 2.7 (2.2,3.3) | 95 | 3.3 (2.7,4.0) | 1.2 (0.7,2.2) | 88 | 3.1 (2.5,3.8) | 1.2 (0.5,3.0) | 139 | 5.6 (4.8,6.7) | 2.1 (0.6,7.1) |
| Pancreatitis | 136 | 4.6 (3.9,5.5) | 172 | 6.0 (5.1,6.9) | 1.3 (0.8,2.0) | 232 | 8.2 (7.2,9.3) | 1.8 (0.6,4.9) | 382 | 15.7 (14.2,17.3) | 3.3 (1.0,10.8) |
| ESRD | 65 | 2.2 (1.7,2.8) | 81 | 2.8 (2.2,3.5) | 1.3 (0.7,2.4) | 123 | 4.3 (3.6,5.1) | 2.0 (0.8,5.1) | 115 | 4.7 (3.9,5.6) | 2.1 (0.4,10.9) |
| AKD | 332 | 11.4 (10.2,12.6) | 386 | 13.5 (12.2,14.9) | 1.2 (0.9,1.6) | 678 | 24.4 (22.6,26.3) | 2.2 (1.4,3.3) | 1,065 | 44.8 (42.1,47.5) | 3.9 (1.7,8.6) |
| All-cause mortality | 9,401 | 318.0 (311.6,324.5) | 13,725 | 474.8 (466.9,482.8) | 1.5 (1.4,1.6) | 28,083 | 989.8 (978.3,1001.5) | 3.1 (2.9,3.3) | 51,341 | 2079.0 (2061.1,2097.0) | 6.4 (5.7,7.1) |

Note: : Major CVD: composite outcomes of heart failure, stroke and coronary artery disease; AF: Atrial fibrillation; ACD: Acute coronary disorder; DVT: Deep vein thrombosis; CPD: Chronic pulmonary disease; SLD: Severe liver disease; ESRD: End stage renal disease; AKD: Acute kidney disease; NA: Not available due to insufficient number. Hazard Ratio was obtained by Cox regression adjusted with weighting.

**Supplementary Table 11. Hazard ratio of post-acute COVID-19 composite outcomes compared to the control groups in fully vaccination subgroup.**

|  | **Control** | | **Mild** | | | **Severe** | | | **Critical** | | |
| --- | --- | --- | --- | --- | --- | --- | --- | --- | --- | --- | --- |
|  | **Events** | **Incidence rate**  **(per 100,000 person per year)** | **Events** | **Incidence rate**  **(per 100,000 person per year)** | **HR** | **Events** | **Incidence rate**  **(per 100,000 person per year)** | **HR** | **Events** | **Incidence rate**  **(per 100,000 person per year)** | **HR** |
| **Age ≤40** |  |  |  |  |  |  |  |  |  |  |  |
| Major CVD | 121 | 3.5 (2.9,4.2) | 124 | 3.6 (3.0,4.3) | 1.0 (0.7,1.6) | 176 | 4.8 (4.1,5.6) | 1.4 (0.4,4.5) | 0 | NA | NA |
| Carditis | 44 | 1.3 (1.0,1.7) | 34 | 1.0 (0.7,1.3) | 0.8 (0.3,1.7) | 0 | NA | NA | 0 | NA | NA |
| AF | 11 | 0.3 (0.2,0.5) | 9 | 0.3 (0.1,0.5) | 0.9 (0.2,4.1) | 74 | 2.0 (1.6,2.5) | 6.6 (0.8,54.2) | 0 | NA | NA |
| Flutter | 4 | 0.1 (0.0,0.3) | 5 | 0.1 (0.0,0.3) | 1.2 (0.1,11.5) | 74 | 2.0 (1.6,2.5) | 17.3 (1.7,172.1) | 0 | NA | NA |
| ACD | 20 | 0.6 (0.4,0.9) | 13 | 0.4 (0.2,0.6) | 0.7 (0.2,2.3) | 0 | NA | NA | 0 | NA | NA |
| DVT | 13 | 0.4 (0.2,0.6) | 10 | 0.3 (0.1,0.5) | 0.7 (0.2,3.3) | 0 | NA | NA | 0 | NA | NA |
| CPD | 90 | 2.6 (2.1,3.2) | 72 | 2.1 (1.6,2.6) | 0.8 (0.5,1.4) | 248 | 6.9 (6.0,7.8) | 2.6 (1.0,7.3) | 0 | NA | NA |
| SLD | 1 | 0.0 (0.0,0.2) | 0 | NA | NA | 56 | 1.5 (1.2,2.0) | 40.1 (2.5,645.2) | 353 | 10.9 (9.8,12.0) | 284.1 (17.5,4,607.0) |
| Pancreatitis | 24 | 0.7 (0.4,1.0) | 18 | 0.5 (0.3,0.8) | 0.8 (0.3,2.2) | 0 | NA | NA | 0 | NA | NA |
| ESRD | 8 | 0.2 (0.1,0.4) | 0 | NA | NA | 0 | NA | NA | 0 | NA | NA |
| AKD | 8 | 0.2 (0.1,0.4) | 5 | 0.2 (0.1,0.3) | 0.7 (0.1,5.4) | 53 | 1.5 (1.1,1.9) | 6.3 (0.8,52.8) | 0 | NA | NA |
| All-cause mortality | 140 | 4.1 (3.4,4.8) | 83 | 2.4 (1.9,3.0) | 0.6 (0.4,1.0) | 252 | 6.9 (6.1,7.8) | 1.5 (0.6,4.3) | 2,305 | 70.8 (67.9,73.7) | 18.3 (3.7,89.7) |
| **Age (40-65)** |  |  |  |  |  |  |  |  |  |  |  |
| Major CVD | 1,516 | 41.0 (39.0,43.2) | 1,519 | 40.3 (38.3,42.4) | 1.0 (0.9,1.1) | 1,289 | 32.4 (30.6,34.2) | 0.8 (0.5,1.2) | 2,449 | 70.5 (67.8,73.4) | 1.7 (0.5,5.5) |
| Carditis | 18 | 0.5 (0.3,0.7) | 23 | 0.6 (0.4,0.9) | 1.3 (0.5,3.5) | 71 | 1.7 (1.3,2.1) | 3.8 (0.5,29.3) | 948 | 25.6 (24.0,27.2) | 54.3 (9.5,310.2) |
| AF | 145 | 3.8 (3.2,4.4) | 190 | 4.9 (4.2,5.6) | 1.3 (0.9,1.9) | 201 | 4.9 (4.2,5.6) | 1.3 (0.4,4.0) | 70 | 1.9 (1.5,2.4) | 0.5 (0.1,3.6) |
| Flutter | 28 | 0.7 (0.5,1.1) | 34 | 0.9 (0.6,1.2) | 1.2 (0.5,2.7) | 0 | NA | NA | 0 | NA | NA |
| ACD | 531 | 13.9 (12.8,15.1) | 473 | 12.1 (11.1,13.3) | 0.9 (0.7,1.1) | 711 | 17.2 (16.0,18.5) | 1.3 (0.7,2.3) | 816 | 22.3 (20.8,23.8) | 1.6 (0.2,11.4) |
| DVT | 50 | 1.3 (1.0,1.7) | 50 | 1.3 (0.9,1.6) | 1.0 (0.5,2.0) | 61 | 1.5 (1.1,1.9) | 1.2 (0.2,8.8) | 0 | NA | NA |
| CPD | 123 | 3.2 (2.7,3.8) | 188 | 4.9 (4.2,5.6) | 1.5 (1.0,2.2) | 108 | 2.7 (2.2,3.2) | 0.9 (0.2,3.5) | 469 | 12.8 (11.7,14.0) | 4.0 (0.6,28.5) |
| SLD | 46 | 1.2 (0.9,1.6) | 49 | 1.2 (0.9,1.6) | 1.0 (0.5,2.1) | 80 | 1.9 (1.5,2.4) | 1.6 (0.3,7.5) | 0 | NA | NA |
| Pancreatitis | 86 | 2.2 (1.8,2.8) | 50 | 1.3 (0.9,1.7) | 0.6 (0.3,1.1) | 0 | NA | NA | 0 | NA | NA |
| ESRD | 17 | 0.4 (0.3,0.7) | 15 | 0.4 (0.2,0.6) | 0.9 (0.2,3.1) | 66 | 1.6 (1.2,2.0) | 3.8 (0.8,18.1) | 32 | 0.9 (0.6,1.2) | 1.9 (0.3,14.8) |
| AKD | 71 | 1.8 (1.4,2.3) | 63 | 1.6 (1.2,2.0) | 0.9 (0.5,1.6) | 109 | 2.6 (2.2,3.2) | 1.5 (0.4,6.2) | 0 | NA | NA |
| All-cause mortality | 1,164 | 30.2 (28.5,31.9) | 1,070 | 27.2 (25.6,28.9) | 0.9 (0.8,1.1) | 1,855 | 44.5 (42.5,46.6) | 1.5 (1.1,2.1) | 11,365 | 305.9 (300.3,311.5) | 10.2 (5.5,18.8) |
| **Age≥65** |  |  |  |  |  |  |  |  |  |  |  |
| Major CVD | 2,409 | 123.2 (118.4,128.2) | 2,400 | 122.8 (118.0,127.8) | 1.0 (0.9,1.1) | 3,063 | 158.5 (153.0,164.2) | 1.3 (1.0,1.7) | 3,504 | 209.1 (202.2,216.1) | 1.7 (0.9,3.1) |
| Carditis | 35 | 1.5 (1.1,2.1) | 46 | 2.0 (1.5,2.6) | 1.3 (0.6,3.0) | 26 | 1.1 (0.8,1.6) | 0.7 (0.1,5.5) | 0 | NA | NA |
| AF | 489 | 21.7 (19.8,23.7) | 633 | 28.1 (26.0,30.4) | 1.3 (1.0,1.6) | 775 | 34.5 (32.1,36.9) | 1.6 (1.0,2.7) | 837 | 41.6 (38.8,44.5) | 1.9 (0.6,6.1) |
| Flutter | 31 | 1.3 (0.9,1.9) | 48 | 2.1 (1.6,2.8) | 1.5 (0.7,3.4) | 32 | 1.4 (0.9,1.9) | 1.0 (0.1,7.6) | 0 | NA | NA |
| ACD | 783 | 34.2 (31.8,36.6) | 661 | 28.8 (26.7,31.1) | 0.8 (0.7,1.0) | 987 | 43.2 (40.6,45.9) | 1.3 (0.8,1.9) | 750 | 36.6 (34.1,39.3) | 1.1 (0.3,3.7) |
| DVT | 57 | 2.4 (1.8,3.1) | 111 | 4.8 (3.9,5.7) | 2.0 (1.1,3.4) | 144 | 6.2 (5.2,7.2) | 2.5 (0.9,7.5) | 355 | 16.9 (15.2,18.7) | 6.7 (1.5,30.2) |
| CPD | 200 | 8.8 (7.7,10.2) | 253 | 11.2 (9.9,12.6) | 1.3 (0.9,1.8) | 343 | 15.3 (13.8,17.0) | 1.8 (0.9,3.5) | 779 | 39.4 (36.7,42.2) | 4.4 (1.5,12.4) |
| SLD | 58 | 2.5 (1.9,3.2) | 71 | 3.0 (2.4,3.8) | 1.2 (0.6,2.3) | 13 | 0.6 (0.3,0.9) | 0.2 (0.0,1.7) | 0 | NA | NA |
| Pancreatitis | 85 | 3.6 (2.9,4.5) | 114 | 4.9 (4.1,5.9) | 1.3 (0.8,2.3) | 133 | 5.7 (4.8,6.7) | 1.6 (0.5,5.4) | 0 | NA | NA |
| ESRD | 30 | 1.3 (0.9,1.8) | 38 | 1.6 (1.1,2.2) | 1.2 (0.5,3.1) | 126 | 5.4 (4.5,6.4) | 4.2 (1.2,14.0) | 0 | NA | NA |
| AKD | 179 | 7.7 (6.6,8.9) | 205 | 8.8 (7.7,10.1) | 1.1 (0.8,1.7) | 438 | 18.9 (17.2,20.7) | 2.5 (1.3,4.7) | 83 | 4.0 (3.2,5.0) | 0.5 (0.1,3.7) |
| All-cause mortality | 4,100 | 175.1 (169.8,180.5) | 5,239 | 223.6 (217.6,229.7) | 1.3 (1.2,1.4) | 11,612 | 495.0 (486.0,504.0) | 2.9 (2.5,3.2) | 23,382 | 1,104.9 (1,090.8,1,119.1) | 6.2 (5.1,7.5) |

Note : Major CVD: composite outcomes of heart failure, stroke and coronary artery disease; AF: Atrial fibrillation; ACD: Acute coronary disorder; DVT: Deep vein thrombosis; CPD: Chronic pulmonary disease; SLD: Severe liver disease; ESRD: End stage renal disease; AKD: Acute kidney disease; NA: Not available due to insufficient number. Hazard Ratio was obtained by Cox regression adjusted with weighting.

**Supplementary Table 12. Hazard ratio of post-acute COVID-19 composite outcomes compared to the control groups in non-fully vaccination subgroup.**

|  | **Control** | | **Mild** | | | **Severe** | | | **Critical** | | |
| --- | --- | --- | --- | --- | --- | --- | --- | --- | --- | --- | --- |
|  | **Events** | **Incidence rate**  **(per 100,000 person per year)** | **Events** | **Incidence rate**  **(per 100,000 person per year)** | **HR** | **Events** | **Incidence rate**  **(per 100,000 person per year)** | **HR** | **Events** | **Incidence rate**  **(per 100,000 person per year)** | **HR** |
| **Age ≤40** |  |  |  |  |  |  |  |  |  |  |  |
| Major CVD | 57 | 2.4 (1.8,3.1) | 111 | 4.8 (3.9,5.7) | 2.0 (1.1,3.4) | 144 | 6.2 (5.2,7.2) | 2.5 (0.9,7.5) | 355 | 16.9 (15.2,18.7) | 6.7 (1.5,30.2) |
| Carditis | 4 | 0.2 (0.0,0.4) | 12 | 0.5 (0.3,0.9) | 3.4 (0.6,20.4) | 116 | 5.0 (4.1,5.9) | 32.4 (5.4,195.1) | 0 | NA | NA |
| AF | 17 | 0.7 (0.5,1.2) | 12 | 0.5 (0.3,0.8) | 0.7 (0.2,3.0) | 87 | 3.8 (3.0,4.6) | 5.1 (1.4,18.8) | 0 | NA | NA |
| Flutter | 3 | 0.1 (0.0,0.3) | 6 | 0.3 (0.1,0.6) | 2.5 (0.2,27.4) | 0 | NA | NA | 0 | NA | NA |
| ACD | 7 | 0.3 (0.1,0.6) | 6 | 0.3 (0.1,0.6) | 0.8 (0.1,6.8) | 0 | NA | NA | 0 | NA | NA |
| DVT | 200 | 8.8 (7.7,10.2) | 253 | 11.2 (9.9,12.6) | 1.3 (0.9,1.8) | 343 | 15.3 (13.8,17.0) | 1.8 (0.9,3.5) | 779 | 39.4 (36.7,42.2) | 4.4 (1.5,12.4) |
| CPD | 4,100 | 175.1 (169.8,180.5) | 5,239 | 223.6 (217.6,229.7) | 1.3 (1.2,1.4) | 11,612 | 495.0 (486.0,504.0) | 2.9 (2.5,3.2) | 23,382 | 1,104.9 (1,090.8,1,119.1) | 6.2 (5.1,7.5) |
| SLD | 57 | 2.4 (1.8,3.1) | 111 | 4.8 (3.9,5.7) | 2.0 (1.1,3.4) | 144 | 6.2 (5.2,7.2) | 2.5 (0.9,7.5) | 355 | 16.9 (15.2,18.7) | 6.7 (1.5,30.2) |
| Pancreatitis | 4 | 0.2 (0.0,0.4) | 12 | 0.5 (0.3,0.9) | 3.4 (0.6,20.4) | 116 | 5.0 (4.1,5.9) | 32.4 (5.4,195.1) | 0 | NA | NA |
| ESRD | 17 | 0.7 (0.5,1.2) | 12 | 0.5 (0.3,0.8) | 0.7 (0.2,3.0) | 87 | 3.8 (3.0,4.6) | 5.1 (1.4,18.8) | 0 | NA | NA |
| AKD | 3 | 0.1 (0.0,0.3) | 6 | 0.3 (0.1,0.6) | 2.5 (0.2,27.4) | 0 | NA | NA | 0 | NA | NA |
| All-cause mortality | 7 | 0.3 (0.1,0.6) | 6 | 0.3 (0.1,0.6) | 0.8 (0.1,6.8) | 0 | NA | NA | 0 | NA | NA |
| **Age (40-65)** | 200 | 8.8 (7.7,10.2) | 253 | 11.2 (9.9,12.6) | 1.3 (0.9,1.8) | 343 | 15.3 (13.8,17.0) | 1.8 (0.9,3.5) | 779 | 39.4 (36.7,42.2) | 4.4 (1.5,12.4) |
| Major CVD | 58 | 2.5 (1.9,3.2) | 71 | 3.0 (2.4,3.8) | 1.2 (0.6,2.3) | 13 | 0.6 (0.3,0.9) | 0.2 (0.0,1.7) | 0 | NA | NA |
| Carditis | 85 | 3.6 (2.9,4.5) | 114 | 4.9 (4.1,5.9) | 1.3 (0.8,2.3) | 133 | 5.7 (4.8,6.7) | 1.6 (0.5,5.4) | 0 | NA | NA |
| AF | 30 | 1.3 (0.9,1.8) | 38 | 1.6 (1.1,2.2) | 1.2 (0.5,3.1) | 126 | 5.4 (4.5,6.4) | 4.2 (1.2,14.0) | 0 | NA | NA |
| Flutter | 179 | 7.7 (6.6,8.9) | 205 | 8.8 (7.7,10.1) | 1.1 (0.8,1.7) | 438 | 18.9 (17.2,20.7) | 2.5 (1.3,4.7) | 83 | 4.0 (3.2,5.0) | 0.5 (0.1,3.7) |
| ACD | 4,100 | 175.1 (169.8,180.5) | 5,239 | 223.6 (217.6,229.7) | 1.3 (1.2,1.4) | 11,612 | 495.0 (486.0,504.0) | 2.9 (2.5,3.2) | 23,382 | 1,104.9 (1,090.8,1,119.1) | 6.2 (5.1,7.5) |
| DVT | 57 | 2.4 (1.8,3.1) | 111 | 4.8 (3.9,5.7) | 2.0 (1.1,3.4) | 144 | 6.2 (5.2,7.2) | 2.5 (0.9,7.5) | 355 | 16.9 (15.2,18.7) | 6.7 (1.5,30.2) |
| CPD | 200 | 8.8 (7.7,10.2) | 253 | 11.2 (9.9,12.6) | 1.3 (0.9,1.8) | 343 | 15.3 (13.8,17.0) | 1.8 (0.9,3.5) | 779 | 39.4 (36.7,42.2) | 4.4 (1.5,12.4) |
| SLD | 58 | 2.5 (1.9,3.2) | 71 | 3.0 (2.4,3.8) | 1.2 (0.6,2.3) | 13 | 0.6 (0.3,0.9) | 0.2 (0.0,1.7) | 0 | NA | NA |
| Pancreatitis | 85 | 3.6 (2.9,4.5) | 114 | 4.9 (4.1,5.9) | 1.3 (0.8,2.3) | 133 | 5.7 (4.8,6.7) | 1.6 (0.5,5.4) | 0 | NA | NA |
| ESRD | 30 | 1.3 (0.9,1.8) | 38 | 1.6 (1.1,2.2) | 1.2 (0.5,3.1) | 126 | 5.4 (4.5,6.4) | 4.2 (1.2,14.0) | 0 | NA | NA |
| AKD | 179 | 7.7 (6.6,8.9) | 205 | 8.8 (7.7,10.1) | 1.1 (0.8,1.7) | 438 | 18.9 (17.2,20.7) | 2.5 (1.3,4.7) | 83 | 4.0 (3.2,5.0) | 0.5 (0.1,3.7) |
| All-cause mortality | 4,100 | 175.1 (169.8,180.5) | 5,239 | 223.6 (217.6,229.7) | 1.3 (1.2,1.4) | 11,612 | 495.0 (486.0,504.0) | 2.9 (2.5,3.2) | 23,382 | 1,104.9 (1,090.8,1,119.1) | 6.2 (5.1,7.5) |
| **Age≥65** | 57 | 2.4 (1.8,3.1) | 111 | 4.8 (3.9,5.7) | 2.0 (1.1,3.4) | 144 | 6.2 (5.2,7.2) | 2.5 (0.9,7.5) | 355 | 16.9 (15.2,18.7) | 6.7 (1.5,30.2) |
| Major CVD | 4 | 0.2 (0.0,0.4) | 12 | 0.5 (0.3,0.9) | 3.4 (0.6,20.4) | 116 | 5.0 (4.1,5.9) | 32.4 (5.4,195.1) | 0 | NA | NA |
| Carditis | 17 | 0.7 (0.5,1.2) | 12 | 0.5 (0.3,0.8) | 0.7 (0.2,3.0) | 87 | 3.8 (3.0,4.6) | 5.1 (1.4,18.8) | 0 | NA | NA |
| AF | 3 | 0.1 (0.0,0.3) | 6 | 0.3 (0.1,0.6) | 2.5 (0.2,27.4) | 0 | NA | NA | 0 | NA | NA |
| Flutter | 7 | 0.3 (0.1,0.6) | 6 | 0.3 (0.1,0.6) | 0.8 (0.1,6.8) | 0 | NA | NA | 0 | NA | NA |
| ACD | 200 | 8.8 (7.7,10.2) | 253 | 11.2 (9.9,12.6) | 1.3 (0.9,1.8) | 343 | 15.3 (13.8,17.0) | 1.8 (0.9,3.5) | 779 | 39.4 (36.7,42.2) | 4.4 (1.5,12.4) |
| DVT | 58 | 2.5 (1.9,3.2) | 71 | 3.0 (2.4,3.8) | 1.2 (0.6,2.3) | 13 | 0.6 (0.3,0.9) | 0.2 (0.0,1.7) | 0 | NA | NA |
| CPD | 57 | 2.4 (1.8,3.1) | 111 | 4.8 (3.9,5.7) | 2.0 (1.1,3.4) | 144 | 6.2 (5.2,7.2) | 2.5 (0.9,7.5) | 355 | 16.9 (15.2,18.7) | 6.7 (1.5,30.2) |
| SLD | 4 | 0.2 (0.0,0.4) | 12 | 0.5 (0.3,0.9) | 3.4 (0.6,20.4) | 116 | 5.0 (4.1,5.9) | 32.4 (5.4,195.1) | 0 | NA | NA |
| Pancreatitis | 17 | 0.7 (0.5,1.2) | 12 | 0.5 (0.3,0.8) | 0.7 (0.2,3.0) | 87 | 3.8 (3.0,4.6) | 5.1 (1.4,18.8) | 0 | NA | NA |
| ESRD | 3 | 0.1 (0.0,0.3) | 6 | 0.3 (0.1,0.6) | 2.5 (0.2,27.4) | 0 | NA | NA | 0 | NA | NA |
| AKD | 7 | 0.3 (0.1,0.6) | 6 | 0.3 (0.1,0.6) | 0.8 (0.1,6.8) | 0 | NA | NA | 0 | NA | NA |
| All-cause mortality | 200 | 8.8 (7.7,10.2) | 253 | 11.2 (9.9,12.6) | 1.3 (0.9,1.8) | 343 | 15.3 (13.8,17.0) | 1.8 (0.9,3.5) | 779 | 39.4 (36.7,42.2) | 4.4 (1.5,12.4) |

Note: AF: Atrial fibrillation; ACD: Acute coronary disorder; DVT: Deep vein thrombosis; CPD: Chronic pulmonary disease; SLD: Severe liver disease; ESRD: End stage renal disease; AKD: Acute kidney disease; NA: Not available due to insufficient number.

Hazard Ratio was obtained by Cox regression adjusted with weighting.

**Supplementary Table 13. Hazard ratio of acute COVID-19 composite outcomes compared to the control groups** **(index date as seven days after COVID-19 infection and possible ICU admission).**

|  | **Control** | | **Mild** | | | **Severe** | | | **Critical** | | |
| --- | --- | --- | --- | --- | --- | --- | --- | --- | --- | --- | --- |
|  | **Events** | **Incidence rate**  **(per 100,000 person per day)** | **Events** | **Incidence rate**  **(per 100,000 person per day)** | **HR** | **Events** | **Incidence rate**  **(per 100,000 person per day)** | **HR** | **Events** | **Incidence rate**  **(per 100,000 person per day)** | **HR** |
| **Age ≤40** |  |  |  |  |  |  |  |  |  |  |  |
| Major CVD | 16 | 45.74 (26.73,72.30) | 7 | 80.03 (32.87,152.53) | 1.7 (0.7,4.1) | 1 | 222.38 (43.99,1,011.98) | 4.9 (0.6,36.8) | 1 | 1,704.19 (73.77,10,738.09) | 37.2 (4.9,281.8) |
| Carditis | 7 | 20.65 (10.08,42.07) | 2 | 22.53 (2.82,64.96) | 1.1 (0.2,5.3) | 1 | 230.30 (43.91,1,010.08) | 11.2 (1.4,90.7) | 0 | NA | NA |
| AF | 2 | 5.51 (0.71,16.25) | 1 | 9.87 (0.30,42.99) | 1.8 (0.2,19.8) | 0 | NA | NA | 0 | NA | NA |
| Flutter | 0 | 0.00 (0.00,10.75) | 1 | 9.87 (0.30,42.98) | NA | 0 | NA | NA | 0 | NA | NA |
| CAD | 4 | 12.36 (4.74,29.87) | 3 | 29.92 (7.21,84.22) | 9.4 (1.9,47.2) | 0 | NA | NA | 0 | NA | NA |
| DVT | 2 | 5.87 (1.80,21.07) | 2 | 21.19 (2.82,64.93) | 3.6 (0.5,25.7) | 0 | NA | NA | 0 | NA | NA |
| CPD | 10 | 29.45 (14.13,50.34) | 13 | 156.15 (90.23,262.08) | 5.3 (2.3,12.2) | 1 | 208.71 (44.62,1,026.50) | 7.1 (0.9,55.4) | 0 | NA | NA |
| SLD | 0 | NA | 0 | NA | NA | 0 | NA | NA | 0 | 0.00 (0.00,10,665.62) | NA |
| Pancreatitis | 6 | 18.07 (8.21,38.09) | 4 | 50.91 (18.92,119.37) | 2.8 (0.9,9.2) | 0 | NA | NA | 2 | 4,884.58 (704.22,16,199.59) | NA |
| ESRD | 1 | 2.71 (0.07,10.75) | 0 | NA | NA | 0 | NA | NA | 0 | NA | NA |
| AKD | 2 | 6.05 (1.80,21.07) | 0 | NA | NA | 0 | NA | NA | 0 | NA | NA |
| All-cause mortality | 46 | 134.75 (100.67,178.86) | 12 | 140.34 (80.65,244.22) | 1.0 (0.5,2.0) | 19 | 3,466.86 (2,212.63,5,373.94) | 25.7 (15.1,43.7) | 10 | 29,266.21 (15,855.53,53,101.56) | 217.0 (109.1,431.7) |
| **Age (40-65)** |  |  |  |  |  |  |  |  |  |  |  |
| Major CVD | 318 | 0.08 (0.07,0.09) | 146 | 0.16 (0.14,0.19) | 2.0 (1.6,2.4) | 36 | 0.53 (0.37,0.72) | 6.6 (4.5,9.6) | 18 | 2.95 (1.76,4.49) | 36.3 (19.4,67.9) |
| Carditis | 8 | 0.00 (0.00,0.00) | 2 | 0.00 (0.00,0.01) | 0.9 (0.2,4.5) | 0 | NA | NA | 0 | NA | NA |
| AF | 30 | 0.01 (0.01,0.01) | 10 | 0.01 (0.01,0.02) | 1.4 (0.6,2.9) | 3 | 0.04 (0.02,0.12) | 6.0 (1.9,18.6) | 0 | NA | NA |
| Flutter | 5 | 0.00 (0.00,0.00) | 2 | 0.00 (0.00,0.01) | 1.5 (0.3,8.0) | 0 | NA | NA | 0 | NA | NA |
| CAD | 143 | 0.04 (0.03,0.04) | 52 | 0.06 (0.04,0.07) | 1.6 (1.1,2.2) | 12 | 0.17 (0.09,0.28) | 4.7 (2.5,8.7) | 10 | 1.64 (0.87,2.92) | 45.8 (20.7,101.1) |
| DVT | 5 | 0.00 (0.00,0.00) | 6 | 0.01 (0.00,0.01) | 5.1 (1.4,18.9) | 1 | 0.02 (0.00,0.08) | 13.0 (1.5,111.2) | 0 | NA | NA |
| CPD | 24 | 0.01 (0.00,0.01) | 16 | 0.02 (0.01,0.03) | 2.9 (1.6,5.4) | 2 | 0.03 (0.01,0.10) | 5.1 (1.5,17.1) | 3 | 0.42 (0.10,1.13) | 71.3 (9.6,527.3) |
| SLD | 10 | 0.00 (0.00,0.00) | 6 | 0.01 (0.00,0.01) | 2.6 (0.9,7.2) | 2 | 0.03 (0.01,0.10) | 13.3 (3.3,53.0) | 0 | 0.00 (0.00,0.57) | NA |
| Pancreatitis | 15 | 0.00 (0.00,0.01) | 9 | 0.01 (0.00,0.02) | 2.5 (1.0,6.1) | 2 | 0.03 (0.01,0.10) | 8.8 (1.9,40.3) | 1 | 0.22 (0.04,0.88) | 60.9 (8.1,459.9) |
| ESRD | 1 | 0.00 (0.00,0.00) | 1 | 0.00 (0.00,0.00) | 4.2 (0.3,67.6) | 0 | NA | NA | 0 | NA | NA |
| AKD | 9 | 0.00 (0.00,0.00) | 11 | 0.01 (0.01,0.02) | 5.2 (2.1,13.0) | 2 | 0.03 (0.00,0.08) | 12.0 (2.4,60.4) | 0 | NA | NA |
| All-cause mortality | 480 | 0.12 (0.11,0.13) | 241 | 0.25 (0.22,0.28) | 2.2 (1.8,2.6) | 125 | 1.77 (1.48,2.10) | 15.1 (12.6,18.3) | 109 | 16.69 (13.76,20.05) | 142.4 (112.4,180.3) |
| **Age≥65** |  |  |  |  |  |  |  |  |  |  |  |
| Major CVD | 635 | 0.26 (0.24,0.28) | 386 | 0.83 (0.75,0.91) | 3.2 (2.8,3.7) | 149 | 2.04 (1.74,2.39) | 7.9 (6.5,9.7) | 86 | 4.06 (3.28,4.99) | 15.4 (10.6,22.4) |
| Carditis | 8 | 0.00 (0.00,0.01) | 3 | 0.00 (0.00,0.01) | 1.8 (0.5,6.8) | 1 | 0.01 (0.00,0.04) | 3.2 (0.4,25.9) | 0 | NA | NA |
| AF | 108 | 0.04 (0.03,0.05) | 68 | 0.12 (0.10,0.15) | 3.3 (2.4,4.5) | 17 | 0.20 (0.12,0.31) | 5.3 (3.1,9.0) | 6 | 0.25 (0.11,0.51) | 6.6 (3.0,14.8) |
| Flutter | 12 | 0.00 (0.00,0.01) | 6 | 0.01 (0.00,0.02) | 2.4 (0.9,6.9) | 0 | NA | NA | 0 | NA | NA |
| CAD | 253 | 0.09 (0.08,0.10) | 128 | 0.24 (0.20,0.29) | 2.7 (2.1,3.3) | 59 | 0.71 (0.55,0.91) | 7.7 (5.7,10.4) | 40 | 1.61 (1.15,2.14) | 17.2 (11.6,25.6) |
| DVT | 16 | 0.01 (0.00,0.01) | 17 | 0.03 (0.02,0.05) | 5.5 (2.7,11.1) | 6 | 0.07 (0.03,0.14) | 12.5 (5.3,29.5) | 6 | 0.21 (0.08,0.43) | 38.1 (13.1,111.3) |
| CPD | 45 | 0.02 (0.01,0.02) | 38 | 0.07 (0.05,0.09) | 4.4 (2.8,6.9) | 8 | 0.09 (0.04,0.17) | 5.9 (3.0,11.7) | 4 | 0.17 (0.06,0.40) | 10.6 (3.1,36.0) |
| SLD | 9 | 0.00 (0.00,0.01) | 11 | 0.02 (0.01,0.03) | 6.0 (2.4,15.3) | 2 | 0.03 (0.01,0.08) | 8.1 (2.1,31.1) | 0 | 0.01 (0.00,0.14) | 3.1 (0.4,24.0) |
| Pancreatitis | 27 | 0.01 (0.01,0.01) | 13 | 0.02 (0.01,0.04) | 2.6 (1.3,5.3) | 3 | 0.03 (0.01,0.08) | 3.5 (0.5,22.5) | 6 | 0.21 (0.08,0.43) | 22.9 (7.8,66.8) |
| ESRD | 14 | 0.00 (0.00,0.01) | 8 | 0.01 (0.01,0.03) | 2.9 (1.1,7.7) | 4 | 0.05 (0.02,0.11) | 10.2 (3.9,26.6) | 2 | 0.09 (0.02,0.27) | 18.7 (7.3,47.6) |
| AKD | 67 | 0.02 (0.02,0.03) | 47 | 0.08 (0.06,0.11) | 3.7 (2.5,5.5) | 18 | 0.20 (0.12,0.30) | 8.8 (5.4,14.4) | 11 | 0.42 (0.24,0.75) | 18.6 (8.1,42.7) |
| All-cause mortality | 3,329 | 1.10 (1.06,1.14) | 3,074 | 5.33 (5.14,5.52) | 4.8 (4.6,5.1) | 1,593 | 17.42 (16.58,18.29) | 15.8 (15.0,16.7) | 1,545 | 56.51 (53.76,59.40) | 50.6 (46.8,54.6) |

Note: Major CVD: composite outcomes of heart failure, stroke and coronary artery disease; AF: Atrial fibrillation; ACD: Acute coronary disorder; DVT: Deep vein thrombosis; CPD: Chronic pulmonary disease; SLD: Severe liver disease; ESRD: End stage renal disease; AKD: Acute kidney disease; NA: Not available due to insufficient number. Hazard Ratio was obtained by Cox regression adjusted with weighting.

**Supplementary Table 14. Hazard ratio of post-acute COVID-19 composite outcomes compared to the control groups (index date as seven days after COVID-19 infection and possible ICU admission).**

|  | **Control** | | **Mild** | | | **Severe** | | | **Critical** | | |
| --- | --- | --- | --- | --- | --- | --- | --- | --- | --- | --- | --- |
|  | **Events** | **Incidence rate**  **(per 100,000 person per day)** | **Events** | **Incidence rate**  **(per 100,000 person per day)** | **HR** | **Events** | **Incidence rate**  **(per 100,000 person per day)** | **HR** | **Events** | **Incidence rate**  **(per 100,000 person per day)** | **HR** |
| **Age ≤40** |  |  |  |  |  |  |  |  |  |  |  |
| Major CVD | 229 | 2.9 (2.5,3.3) | 54 | 2.7 (2.1,3.5) | 0.9 (0.7,1.3) | 6 | 4.3 (1.6,8.7) | 1.5 (0.7,3.3) | 0 | NA | NA |
| Carditis | 75 | 0.9 (0.7,1.2) | 17 | 0.9 (0.5,1.3) | 0.9 (0.5,1.5) | 3 | 2.2 (0.5,5.4) | 2.4 (0.7,8.1) | 1 | 12.9 (3.0,70.1) | 13.7 (1.9,98.5) |
| AF | 22 | 0.3 (0.2,0.4) | 8 | 0.4 (0.2,0.8) | 1.5 (0.7,3.5) | 2 | 1.9 (0.5,5.4) | 6.3 (1.6,25.6) | 0 | NA | NA |
| Flutter | 5 | 0.1 (0.0,0.1) | 3 | 0.1 (0.0,0.4) | 2.0 (0.5,8.5) | 1 | 0.7 (0.0,2.8) | 10.2 (1.2,87.6) | 0 | NA | NA |
| CAD | 65 | 0.8 (0.6,1.0) | 13 | 0.7 (0.4,1.1) | 1.8 (0.8,4.4) | 2 | NA | NA | 0 | NA | NA |
| DVT | 20 | 0.2 (0.2,0.4) | 6 | 0.3 (0.1,0.7) | 1.3 (0.5,3.0) | 1 | 0.9 (0.2,4.2) | 3.6 (0.5,27.6) | 0 | NA | NA |
| CPD | 322 | 4.1 (3.7,4.5) | 73 | 3.7 (2.9,4.6) | 0.9 (0.7,1.2) | 11 | 8.3 (4.2,14.0) | 2.1 (1.2,3.6) | 0 | NA | NA |
| SLD | 16 | 0.2 (0.1,0.3) | 4 | 0.2 (0.1,0.4) | 0.9 (0.3,2.8) | 1 | 0.6 (0.0,2.8) | 2.8 (0.4,21.0) | 0 | NA | NA |
| Pancreatitis | 47 | 0.6 (0.4,0.8) | 12 | 0.6 (0.3,1.0) | 1.0 (0.5,1.9) | 1 | NA | NA | 0 | NA | NA |
| ESRD | 12 | 0.1 (0.1,0.2) | 2 | 0.1 (0.0,0.3) | 0.5 (0.1,4.1) | 1 | NA | NA | 0 | 0.0 (0.0,46.4) | 0.0 (0.0,0.0) |
| AKD | 22 | 0.3 (0.2,0.4) | 5 | 0.3 (0.1,0.6) | 1.0 (0.4,2.7) | 1 | 0.4 (0.0,2.8) | 1.4 (0.2,10.3) | 0 | NA | NA |
| All-cause mortality | 279 | 3.5 (3.1,3.9) | 55 | 2.7 (2.1,3.5) | 0.8 (0.6,1.1) | 9 | 6.9 (3.6,12.7) | 2.0 (1.1,3.8) | 4 | 50.2 (20.4,128.4) | 14.3 (5.4,37.8) |
| **Age (40-65)** |  |  |  |  |  |  |  |  |  |  |  |
| Major CVD | 3,699 | 40.1 (38.8,41.4) | 860 | 40.1 (37.5,42.9) | 1.0 (0.9,1.1) | 88 | 52.8 (42.6,64.8) | 1.3 (1.1,1.7) | 7 | 58.7 (27.2,113.7) | 1.5 (0.7,2.9) |
| Carditis | 56 | 0.6 (0.4,0.8) | 9 | 0.4 (0.2,0.8) | 0.7 (0.4,1.4) | 1 | 0.8 (0.1,3.2) | 1.3 (0.3,6.1) | 2 | 12.2 (1.8,41.2) | 21.1 (5.4,83.0) |
| AF | 360 | 3.7 (3.4,4.1) | 114 | 5.1 (4.2,6.1) | 1.4 (1.1,1.7) | 7 | 4.2 (2.0,8.3) | 1.1 (0.5,2.3) | 0 | NA | NA |
| Flutter | 55 | 0.6 (0.4,0.7) | 19 | 0.8 (0.5,1.3) | 1.4 (0.8,2.6) | 0 | NA | NA | 0 | NA | NA |
| CAD | 1,677 | 17.8 (17.0,18.7) | 408 | 18.6 (16.9,20.5) | 1.0 (0.9,1.2) | 47 | 27.9 (20.8,36.8) | 1.6 (1.2,2.2) | 4 | 30.9 (12.3,77.8) | 1.7 (0.7,4.5) |
| DVT | 132 | 1.4 (1.1,1.6) | 31 | 1.4 (1.0,2.0) | 1.0 (0.7,1.5) | 3 | 1.9 (0.6,5.1) | 1.4 (0.4,4.7) | 0 | 0.0 (0.0,27.2) | 0.0 (0.0,0.0) |
| CPD | 324 | 3.4 (3.0,3.8) | 101 | 4.6 (3.8,5.5) | 1.4 (1.1,1.7) | 7 | 4.0 (1.6,7.6) | 1.2 (0.5,2.7) | 0 | NA | NA |
| SLD | 127 | 1.3 (1.1,1.6) | 25 | 1.1 (0.8,1.6) | 0.9 (0.5,1.3) | 4 | 2.5 (0.9,5.9) | 2.0 (0.9,4.3) | 0 | NA | NA |
| Pancreatitis | 182 | 1.9 (1.6,2.2) | 42 | 1.9 (1.4,2.5) | 1.0 (0.7,1.4) | 4 | 2.1 (0.6,5.1) | 1.1 (0.3,3.6) | 1 | 8.4 (1.8,42.0) | 4.4 (0.6,31.8) |
| ESRD | 49 | 0.5 (0.4,0.7) | 14 | 0.6 (0.3,1.0) | 1.2 (0.6,2.3) | 4 | 2.2 (0.6,5.0) | 4.1 (1.5,11.2) | 0 | NA | NA |
| AKD | 157 | 1.6 (1.4,1.9) | 40 | 1.8 (1.3,2.4) | 1.1 (0.8,1.6) | 2 | 1.2 (0.4,4.2) | 0.7 (0.3,2.1) | 0 | 0.8 (0.2,27.5) | 0.5 (0.1,3.3) |
| All-cause mortality | 2,758 | 28.6 (27.5,29.6) | 727 | 32.4 (30.1,34.8) | 1.1 (1.0,1.2) | 149 | 85.5 (72.4,99.9) | 3.1 (2.6,3.6) | 33 | 245.1 (173.1,340.7) | 8.4 (6.0,11.8) |
| **Age≥65** |  |  |  |  |  |  |  |  |  |  |  |
| Major CVD | 7,801 | 135.2 (132.2,138.2) | 1,657 | 155.2 (147.9,162.8) | 1.1 (1.1,1.2) | 307 | 201.8 (180.3,225.4) | 1.5 (1.3,1.7) | 70 | 214.7 (167.6,268.3) | 1.6 (1.1,2.3) |
| Carditis | 91 | 1.3 (1.0,1.6) | 25 | 1.9 (1.2,2.7) | 1.4 (0.9,2.3) | 3 | 1.3 (0.3,3.8) | 1.0 (0.3,3.2) | 0 | 0.0 (0.0,8.8) | 0.0 (0.0,0.0) |
| AF | 1,526 | 22.5 (21.4,23.7) | 385 | 30.6 (27.7,33.8) | 1.4 (1.2,1.5) | 66 | 36.2 (28.2,45.8) | 1.6 (1.2,2.1) | 7 | 16.8 (7.2,33.2) | 0.7 (0.4,1.5) |
| Flutter | 111 | 1.6 (1.3,1.9) | 37 | 2.8 (2.0,3.8) | 1.8 (1.2,2.6) | 1 | 0.8 (0.1,3.0) | 0.5 (0.1,1.6) | 1 | 1.2 (0.1,9.0) | 0.8 (0.2,3.2) |
| CAD | 3,077 | 2.8 (2.0,3.8) | 674 | 56.0 (51.9,60.4) | 1.2 (1.1,1.3) | 131 | 75.3 (63.1,89.0) | 1.6 (1.3,1.9) | 23 | 59.7 (38.0,86.9) | 1.3 (0.8,2.0) |
| DVT | 261 | 3.7 (3.3,4.2) | 68 | 5.2 (4.1,6.5) | 1.4 (1.0,1.9) | 13 | 6.8 (3.7,11.1) | 1.8 (1.2,2.9) | 6 | 14.9 (6.8,31.4) | 4.0 (1.2,13.1) |
| CPD | 840 | 12.3 (11.5,13.2) | 209 | 16.6 (14.5,19.0) | 1.3 (1.1,1.6) | 50 | 27.8 (21.1,36.6) | 2.3 (1.7,3.0) | 8 | 20.3 (8.8,36.9) | 1.7 (0.9,3.2) |
| SLD | 148 | 2.1 (1.8,2.5) | 27 | 2.1 (1.4,3.0) | 1.0 (0.7,1.5) | 3 | 1.4 (0.3,3.8) | 0.7 (0.3,1.5) | 3 | 7.6 (2.6,20.9) | 3.6 (1.0,13.4) |
| Pancreatitis | 316 | 4.5 (4.0,5.0) | 68 | 5.2 (4.1,6.5) | 1.2 (0.9,1.5) | 14 | 7.5 (4.4,12.4) | 1.7 (1.0,2.9) | 5 | 12.9 (5.3,28.2) | 2.9 (1.3,6.3) |
| ESRD | 152 | 2.1 (1.8,2.5) | 28 | 2.2 (1.5,3.1) | 1.0 (0.7,1.5) | 10 | 5.0 (2.5,9.0) | 2.4 (1.3,4.3) | 2 | 4.3 (0.6,13.3) | 2.0 (0.8,4.7) |
| AKD | 756 | 10.8 (10.0,11.6) | 165 | 12.6 (10.8,14.7) | 1.2 (1.0,1.4) | 34 | 18.2 (13.0,25.4) | 1.7 (1.3,2.3) | 22 | 54.3 (34.2,79.7) | 5.0 (3.0,8.5) |
| All-cause mortality | 15,131 | 213.7 (210.3,217.1) | 4,248 | 321.2 (311.6,331.0) | 1.5 (1.4,1.6) | 1,234 | 647.9 (612.4,684.7) | 3.1 (2.9,3.2) | 499 | 1,189.3 (1,089.2,1,298.0) | 5.4 (4.9,6.0) |

Note: Major CVD: composite outcomes of heart failure, stroke and coronary artery disease; AF: Atrial fibrillation; ACD: Acute coronary disorder; DVT: Deep vein thrombosis; CPD: Chronic pulmonary disease; SLD: Severe liver disease; ESRD: End stage renal disease; AKD: Acute kidney disease; NA: Not available due to insufficient number. Hazard Ratio was obtained by Cox regression adjusted with weighting.

**Supplementary Table 15. P-value of acute COVID-19 composite outcomes compared to the control groups.**

|  | Mild | Severe | Critical |
| --- | --- | --- | --- |
| **Age ≤40** |  |  |  |
| Major CVD | 0.201682 | 0.125789 | <0.0001 |
| Heart failure | NA | NA | <0.0001 |
| Carditis | 0.913878 | 0.024052 | 0.001744 |
| Stroke | 0.454104 | 0.006239 | <0.0001 |
| AF | 0.633353 | NA | NA |
| Flutter | <0.0001 | NA | NA |
| CAD | 0.247284 | NA | NA |
| ACD | 0.057994 | <0.0001 | <0.0001 |
| DVT | 0.199241 | NA | NA |
| COPD | <0.0001 | 0.061925 | NA |
| SLD | NA | NA | NA |
| Pancreatitis | 0.087398 | NA | <0.0001 |
| ESRD | NA | NA | <0.0001 |
| AKD | NA | NA | <0.0001 |
| All-cause mortality | 0.906615 | <0.0001 | <0.0001 |
| Age (40-65) |  |  |  |
| Major CVD | <0.0001 | <0.0001 | <0.0001 |
| Heart failure | 0.001029 | 0.618298 | <0.0001 |
| Carditis | 0.943332 | NA | NA |
| Stroke | <0.0001 | <0.0001 | <0.0001 |
| AF | 0.431018 | 0.001970 | NA |
| Flutter | 0.606878 | NA | NA |
| CAD | 0.008979 | 0.000001 | <0.0001 |
| ACS | 0.006475 | 0.000001 | <0.0001 |
| DVT | 0.014715 | 0.019168 | NA |
| COPD | 0.000813 | 0.008477 | <0.0001 |
| SLD | 0.066155 | 0.000249 | 0.000034 |
| Pancreatitis | 0.050364 | 0.005101 | 0.000063 |
| ESRD | 0.308023 | NA | 0.000116 |
| AKD | 0.000458 | 0.002684 | 0.001086 |
| All-cause mortality | <0.0001 | <0.0001 | <0.0001 |
| **Age≥65** |  |  |  |
| Major CVD | <0.0001 | <0.0001 | <0.0001 |
| Heart failure | <0.0001 | <0.0001 | <0.0001 |
| Carditis | 0.349490 | 0.353872 | <0.0001 |
| Stroke | <0.0001 | <0.0001 | <0.0001 |
| AF | <0.0001 | <0.0001 | <0.0001 |
| Flutter | 0.235323 | 0.556851 | 0.204577 |
| CAD | <0.0001 | <0.0001 | <0.0001 |
| ACS | <0.0001 | <0.0001 | <0.0001 |
| DVT | 0.000066 | <0.0001 | <0.0001 |
| COPD | <0.0001 | <0.0001 | <0.0001 |
| SLD | 0.066674 | 0.233121 | <0.0001 |
| Pancreatitis | 0.357517 | <0.0001 | <0.0001 |
| ESRD | 0.001973 | 0.000702 | 0.006757 |
| AKD | 0.000006 | <0.0001 | <0.0001 |
| All-cause mortality | <0.0001 | <0.0001 | <0.0001 |

Note: Major CVD: composite outcomes of heart failure, stroke and coronary artery disease; AF: Atrial fibrillation; CAD: coronary artery disease ACS: Acute coronary disorder; DVT: Deep vein thrombosis; COPD: Chronic pulmonary disease; SLD: Severe liver disease; ESRD: End stage renal disease; AKD: Acute kidney disease; NA: Not available due to insufficient number.

Hazard Ratio was obtained by Cox regression adjusted with weighting.

**Supplementary 16. P-value of post-acute COVID-19 composite outcomes compared to the control groups.**

|  | Mild | Severe | Critical |
| --- | --- | --- | --- |
| **Age≤40** |  |  |  |
| Major CVD | 0.433834 | 0.594427 | NA |
| Heart failure | 0.637950 | NA | NA |
| Carditis | 0.992999 | 0.075770 | 0.005005 |
| Stroke | 0.866846 | 0.060995 | 0.544283 |
| AF | 0.356929 | 0.279791 | NA |
| Flutter | 0.331682 | 0.034109 | NA |
| CAD | 0.382954 | NA | NA |
| ACS | 0.965938 | NA | NA |
| DVT | 0.654319 | 0.167559 | NA |
| COPD | 0.6539827 | 0.0328853 | 0.4549784 |
| SLD | 0.5914271 | 0.1043816 | 0.0021215 |
| Pancreatitis | 0.8376642 | NA | NA |
| ESRD | 0.6826451 | NA | <0.0001 |
| AKD | 0.2852023 | 0.3612602 | NA |
| All-cause mortality | 0.1207717 | 0.0319754 | <0.0001 |
| **Age (40-65)** |  |  |  |
| Major CVD | 0.8711237 | 0.0840817 | 0.1765252 |
| Heart failure | 0.2769528 | 0.4849323 | 0.6145 |
| Carditis | 0.5924522 | 0.4276655 | <0.0001 |
| Stroke | 0.1999133 | 0.595829 | 0.5405415 |
| AF | 0.0194986 | 0.604161 | 0.688424 |
| Flutter | 0.3214013 | 0.4427528 | NA |
| CAD | 0.2435295 | 0.028831 | 0.1269634 |
| ACS | 0.6018153 | 0.1926065 | 0.2733689 |
| DVT | 0.7017527 | 0.3722231 | NA |
| COPD | 0.016599 | 0.7122706 | 0.7063486 |
| SLD | 0.8328493 | 0.2794026 | 0.8068363 |
| Pancreatitis | 0.7772453 | 0.8257107 | 0.0803245 |
| ESRD | 0.8108142 | 0.0199644 | 0.516609 |
| AKD | 0.2795016 | 0.6172936 | 0.085014 |
| All-cause mortality | 0.0084087 | <0.0001 | <0.0001 |
| **Age≥65** |  |  |  |
| Major CVD | <0.0001 | <0.0001 | 0.063977 |
| Heart failure | <0.0001 | <0.0001 | <0.0001 |
| Carditis | 0.562199 | 0.765280 | NA |
| Stroke | 0.014844 | 0.014692 | 0.937196 |
| AF | 0.000003 | 0.000305 | 0.721267 |
| Flutter | 0.019912 | 0.413193 | 0.939078 |
| CAD | 0.000059 | 0.000016 | 0.131602 |
| ACS | 0.000722 | 0.000932 | 0.530952 |
| DVT | 0.0019018 | 0.0016588 | 0.000251 |
| COPD | 0.000519 | 0.000004 | 0.194947 |
| SLD | 0.768788 | 0.732886 | 0.070613 |
| Pancreatitis | 0.076787 | 0.183660 | 0.111603 |
| ESRD | 0.372521 | 0.001496 | 0.728954 |
| AKD | 0.121405 | 0.000007 | 0.000041 |
| All-cause mortality | <0.0001 | <0.0001 | <0.0001 |

Note: Major CVD: composite outcomes of heart failure, stroke and coronary artery disease; AF: Atrial fibrillation; CAD: coronary artery disease ACS: Acute coronary disorder; COPD: Chronic pulmonary disease; SLD: Severe liver disease; ESRD: End stage renal disease; AKD: Acute kidney disease; NA: Not available due to insufficient number.

Hazard Ratio was obtained by Cox regression adjusted with weighting.
